# Facilitators and barriers to the implementation of prehabilitation for frail patients into routine health care: a realist review

**DOI:** 10.1186/s12913-024-10665-1

**Published:** 2024-02-13

**Authors:** Anna Frederike Sontag, Jörn Kiselev, Stefan J Schaller, Claudia Spies, Tanja Rombey

**Affiliations:** 1 Berlin School of Public Health, Charité - Universitätsmedizin Berlin, Charitéplatz 1, 10117 Berlin, Germany; 2 Department for Anesthesiology and Intensive Care Medicine Campus Charité Mitte and Campus Virchow-Klinikum, Charité - Universitätsmedizin Berlin, Corporate Member of Freie Universität Berlin, Humboldt-Universität zu Berlin, and Berlin Institute of Health, Charitéplatz 1, 10117 Berlin, Germany; 3 Department of Anesthesiology and Intensive Care, Klinikum rechts der Isar, Technical University of Munich, School of Medicine, Ismaninger Str.22, 81675 München, Germany; 4 Department of Health Care Management, Technische Universität Berlin, Straße des 17. Juni 135, 10623 Berlin, Germany

**Keywords:** Prehabilitation, Implementation, Frailty, Realist review, Barriers, Facilitators

## Abstract

**Background:**

Despite evidence supporting the effectiveness of prehabilitation as a new preoperative care pathway to optimise perioperative outcomes, its implementation into routine health care is widely pending. Frail patients might particularly benefit from prehabilitation interventions, but facilitating and hindering factors need to be considered in the implementation process. Thus, our aim was to derive a programme theory on what prehabilitation programmes work for frail patients in what circumstances and why.

**Methods:**

Following Pawson’s realist review approach, preliminary programme theories on facilitators and barriers were established. General and topic-specific databases were searched systematically for facilitators and barriers to the implementation of prehabilitation for frail patients. Articles were included if they dealt with multimodal prehabilitation programmes prior to surgery in a frail population and if they contained information on facilitators and barriers during the implementation process in the full text. Based on these articles, refined programme theories were generated.

**Results:**

From 2,609 unique titles, 34 were retained for the realist synthesis. Facilitating factors included the individualisation of prehabilitation programmes to meet the patients’ needs and abilities, multimodality, adaption to the local setting and health care system, endorsement by an ambassador and sharing of responsibilities among a multidisciplinary team. Central barriers for frail patients were transportation, lack of social support, and inadequate, overwhelming information provision.

**Conclusions:**

Implementing prehabilitation as a new care pathway for frail patients requires organisational readiness and adaptability to the local setting. On an individual level, a clear understanding of responsibilities and of the intervention’s goal among patients and providers are necessary. Added attention must be paid to the individualisation to fit the needs and restrictions of frail patients. This makes prehabilitation a resource-intense, but promising intervention for frail surgery patients.

**Trial registration:**

PROSPERO (CRD42022335282).

**Supplementary Information:**

The online version contains supplementary material available at 10.1186/s12913-024-10665-1.

## Contributions to the literature

- Prehabilitation is a new care pathway aiming to make patients fit for an upcoming surgery. Frail patients might particularly benefit from prehabilitation.
- To support future implementation of prehabilitation for frail patients, this realist review looked at what works, for whom, in what circumstances and why based on the existing literature in the field.
- A total of 34 documents were found and used to create refined programme theories on the facilitators and barriers to implementation.
- Based on the review’s findings, we present evidence-based recommendations for stakeholders seeking to implement prehabilitation for frail surgical patients, adding to the existing literature at a meta-level.

## Background

### Rationale for review

Prehabilitation is an intervention prior to surgery that aims to improve modifiable risk factors as well as the overall functional capacity of a patient, thereby increasing the ability to cope with the stress of surgery [1]. It extends the rehabilitation phase to pre-surgery and is thus a way of targeting risk factors that can have an adverse impact on the surgical outcome [2]. Prehabilitation is usually multimodal, meaning different types of therapy such as physiotherapy, occupational therapy, and nutritional counselling, amongst others, are combined to prepare individuals for the upcoming intervention [3]. Evidence suggests that the various modes of preoperative intervention can have a positive effect on postoperative outcomes such as length of stay or postoperative complications [4–7] and it has been postulated that especially high-risk individuals, such as elderly, frail or comorbid patients, might benefit from prehabilitation [8].

Frailty is defined as a multidimensional geriatric syndrome, which displays as individuals being more vulnerable to internal and external stressors along with a lack of individual reserve capacity [9]. Frailty is prevalent among 4 to 59 % of community-dwelling elderly, increasing with age, and is more prevalent among women [10]. Because frailty is associated with surgical morbidity and mortality, but is generally a modifiable risk factor [11], an improvement of the frailty status of patients prior to surgery through prehabilitation can have a positive impact on lives post-surgery [12]. The evidence from randomised trials on prehabilitation for frail patients is currently limited with only a small number of trials completed and published yet, such as Carli et al. 2020 [13] and McIsaac et al. 2022 [14]. Although these trials found no significant effects, prehabilitation reduced the prevalence of (severe) complications [13, 14].

The implementation of prehabilitation into routine care, i.e. the systematic uptake of evidence-based practices into standard practice [15], is still widely pending [16]. One reason for the lagging implementation process may be that prehabilitation is an intervention within a complex adaptive system [17], meaning it depends on the patients’ behaviour and on multidisciplinary cooperation between different health care professions and disciplines. Complex interventions are usually context-specific, and many factors determine whether the implementation will be successful and show results comparable to those observed under clinical trial conditions [17, 18]. When the complex intervention addresses a population with specific needs, like frail patients, even more factors apply due to the complexity of the population itself.

The aim of this realist review was to derive a theory on what prehabilitation programmes work for frail patients in what circumstances and why. Our objectives were to identify facilitators and barriers to the implementation of prehabilitation programmes for frail patients prior to elective surgery, and thereby inform future implementation of prehabilitation into routine care.

## Methods

We chose Pawson’s realist review approach [19] as it is the gold standard method for investigating what “What works, for whom, in what circumstances and why?”. To report the realist review process and findings, the Realist and Meta-narrative Evidence Syntheses: Evolving Standards (RAMESES) publication standards [20, 21] (Appendix A) and Preferred Reporting Items for Systematic Reviews and Meta-Analyses (PRISMA) 2020 Checklist [22, 23] (Appendix B) were used. The review protocol was registered at PROSPERO prior to conducting the systematic literature searches (CRD42022335282). No changes were made to the review process as documented in the registration on PROSPERO.

### Realist review design

Realist reviews present an evidence-based method to derive implementation determinants. Pawson's realist review approach [19] is theory-driven and aims to provide an understanding on the successes and challenges of complex interventions by taking the context and working mechanisms into account [24]. Realist reviews start with creating a preliminary programme theory by investigating the relations between contexts, mechanisms, and outcomes in which a specific intervention or programme is implemented. The preliminary programme theory is then refined based on the evidence. Contexts describe the circumstances, in which an intervention is implemented, whereas mechanisms describe how the intervention will work given the specific context. The outcome results from the context-mechanism interaction. The body of context-mechanism-outcome configurations (CMOC) forms the programme theory.

### Development of preliminary programme theories

To give adequate consideration to the complex nature of prehabilitation [17], preliminary programme theories on prehabilitation were developed in an iterative process without regard for specific target populations. To that end, an initial exploratory literature search on prehabilitation using medical subject headings and free text as search terms was performed primarily in PubMed. This background search was used for familiarisation with the literature and the main concepts of the intervention. These searches as well as backward citation searches aimed to specifically identify publications that reference determinants of successful implementation of prehabilitation.

Two context-mechanism-outcome (CMO) configurations, one for facilitators and one for barriers, were identified from individual articles and documented in detail. These CMO configurations were further condensed into the preliminary programme theories, which formed the basis for testing and refinement throughout the realist review process. The preliminary programme theories were extensively discussed within the multidisciplinary research team that is experienced in both prehabilitation and frailty.

### Searching processes

For the systematic literature search, the databases MEDLINE via Pubmed, Embase via Ovid, Cochrane Library, and PEDro were searched on June 7, 2022. The databases were selected to be complementary and as extensive as possible within the scope of this realist review. Furthermore, forward and backward reference searching was conducted using Google Scholar. For grey literature, ProQuest Dissertations & Theses global was searched to identify relevant dissertations, and as an additional source for grey/non-academic literature, the first one hundred results of a Google search (in private search mode and sorted according to relevance) were screened.

The search strategy was developed for MEDLINE using PubMed and then translated to fit the other databases. The strategy included various term combinations to account for the prehabilitative intervention (e.g., “prehab*” or “preoperative exercise”) as well as the frail patient group (e.g., “frail*” or “geriatric*”). Because facilitators and barriers are not always explicitly named as such or might not be mentioned in the title, abstract or keywords, no search terms targeting these concepts were included in the search. The search strategies can be found in Appendix C*.* All search results from the database searches were imported to and stored in the literature management software EndNote 20 [25].

### Selection and appraisal of documents

The in- and exclusion criteria are detailed in Table 1. Documents were included for full-text screening if they dealt with prehabilitation programmes prior to surgery. To define prehabilitation, we used a slightly modified version of the Gurlit et al. 2019 definition: *“a multidisciplinary approach to the care of patients awaiting surgery and nonsurgical procedures to reduce vulnerability and to increase resilience to periinterventional and postinterventional risks, accelerate and improve outcomes and quality of life, and reduce healthcare costs”* [26]. Originally, this definition includes multimodality, but to widen the scope of this realist review, we decided to also accept unimodal programmes if they went beyond medication or supplement intake. At the title-and-abstract-screening stage, we included all references that appeared to focus on prehabilitation and frailty. At the full-text-screening stage, articles were only included if they actually focused on prehabilitation and frailty and also contained information on any challenges, problems, supportive or helpful factors for the implementation of prehabilitation programmes for frail patients.

**Table 1:** Review inclusion and exclusion criteria

**Inclusion criteria**	
P – population	Frail patients who had to undergo surgery; prehabilitation patients had to include frail individuals; term frail/frailty had to be used in the article, or a structured/standardised frailty assessment had to be conducted, e.g., concept of frailty by Fried et al. [27]
I – intervention	Prehabilitation programme
C – comparator	Experimental studies could include a comparison group, but this was not a condition of inclusion. Observational studies and other article types did not have to include a comparison group.
O – outcomes	Facilitators and barriers to the implementation of prehabilitation for frail patients into routine health care. These can be considered from different perspectives such as the patients, the surgeons, the institutions, or the therapists carrying out the prehabilitation programme.
S – study design	No restriction on study design, includes non-empirical sources, or publication type, i.e., grey literature like dissertations, opinion papers etc.
H – healthcare context	Any healthcare setting that provides prehabilitation to frail patients, including ambulatory, inpatient, or partially inpatient, or community settings. Home-based interventions, including tele-medical interventions, were also included.
**Exclusion criteria**	
- Publication language other than English or German - Study registration records and other documentation (e.g., conference abstracts) of ongoing studies on the (cost-)effectiveness and/or safety of prehabilitation - Programmes that were comprised of medication or supplement intake only as well as mere educational programmes - Prehabilitation programmes prior to chemotherapy or other non-surgical interventions - Articles were excluded if they did not contain information that hints at challenges, problems, supportive or helpful factors for the implementation of prehabilitation programmes for frail patients	

Articles were included regardless of publication type (full-text article, conference abstract) and publication date. Only articles written in English or German (authors’ first language) were included for there were no resources for translating articles. Empirical research of any study design (experimental or observational) or data type (quantitative, qualitative or mixed methods), was considered for inclusion. Grey literature, such as dissertations and theses, and other article types were also included. Study registration records and other documentation (e.g., conference abstracts) of ongoing studies on the (cost-) effectiveness or safety of prehabilitation were excluded since for this evaluation, implementation results were relevant.

The research tool Rayyan was used to remove duplicate records and for screening database results [28]. A randomly selected 10%-sample of the search results was screened by title and abstract by two reviewers (AFS, TR) independently. As an agreement rate of more than 80% between reviewers was achieved after the first 10%-sample, the remaining results were screened by one reviewer, who consulted with members of the review team in case of uncertainty. For full-text screening, a new random 10%-sample of the full texts was selected and screened by the two independent reviewers (AFS, TR) until they achieved sufficient agreement (≥ 80%). An agreement rate of 92% was achieved after screening two 10%-samples (excluding conference abstracts or articles where the full text was not yet available). The remaining full texts were screened by one reviewer, again consulting with members of the review team in case of uncertainty. Screening of full texts was conducted along the above PICOSH scheme, noting the reason for exclusion in order of the acronym (e.g., “population” if the focus was not on frail patients).

### Data extraction

Data was extracted by one reviewer (AFS) using Microsoft Word. The selection of data items represents items the reviewers considered relevant to the implementation process and included:

1. Document type and study design (if applicable)
2. Study description (if applicable): location, study period, sample size, sample characteristics
3. Description of the context: disease focus, surgery type, frailty assessment and description of prehabilitation
4. Quotations on barriers
5. Quotations on facilitators
6. Conflict of interest and funding

### Analysis and synthesis

Included articles were read and re-read to identify their contributions to the refinement of the preliminary programme theories in respect to the target population of frail patients. Particular attention was paid to facilitating factors and barriers identified in the preliminary programme theories. Contributions from the included articles could both be supporting or disconfirming the preliminary programme theories. The analysis was not limited to these preliminary concepts and additional context-mechanism-outcome configurations for facilitators and barriers unique to frail patients could be added. Contributions from the included literature were classified and manually color-coded, then summarised to overarching concepts [29]. Results were presented graphically in tables.

### Relevance and rigour of the included literature

Unlike systematic reviews and meta-analyses, realist reviews do not include standardised quality assessments of the literature, but consider even study fragments and not only studies as a whole when evaluating its quality and relevance [30]. Relevance relates to the contribution each selected study makes to the synthesis of the programme theories [30]. Rigour was not judged using standardised checklists but as a non-standardised judgement of how pieces of evidence within the review are used [30].

In this realist review, only articles that contained information on facilitators and/or barriers were included, making all of them relevant to theory-building. The variation in the quality of information provided in the articles translates to the rigour of the study. Rigour, in this review, was assessed by looking at the type of study design and at the context, in which the insights on facilitators and barriers were gained. We differentiated between insights gained from real life situations, in which prehabilitation was implemented (higher rigour), and artificial study situations (lesser rigour). Similarly, we differentiated between the different study designs. Observational studies in real health system settings as well as qualitative interviews that provide first-hand information on context-specific factors affecting the implementation process were considered of higher rigour than systematic reviews, which provide more generalised information, although both types of information can be helpful in the synthesis process. Information from editorials or opinion pieces, on the other hand, should be considered with more caution as the quality of the information can vary with the expertise of the author, and were thus regarded of less rigour.

## Results

### Preliminary programme theories

Two preliminary programme theories describing five CMOCs for facilitators and five CMOCs for barriers to the implementation of prehabilitation programmes built the basis for the review process (see tables and supporting quotations in Appendix D and E). Amongst others, the CMOCs covered the themes of information provision, patient-centredness, programme adaptability, and multidisciplinary providers.

### Search results

**Fig. 1:**
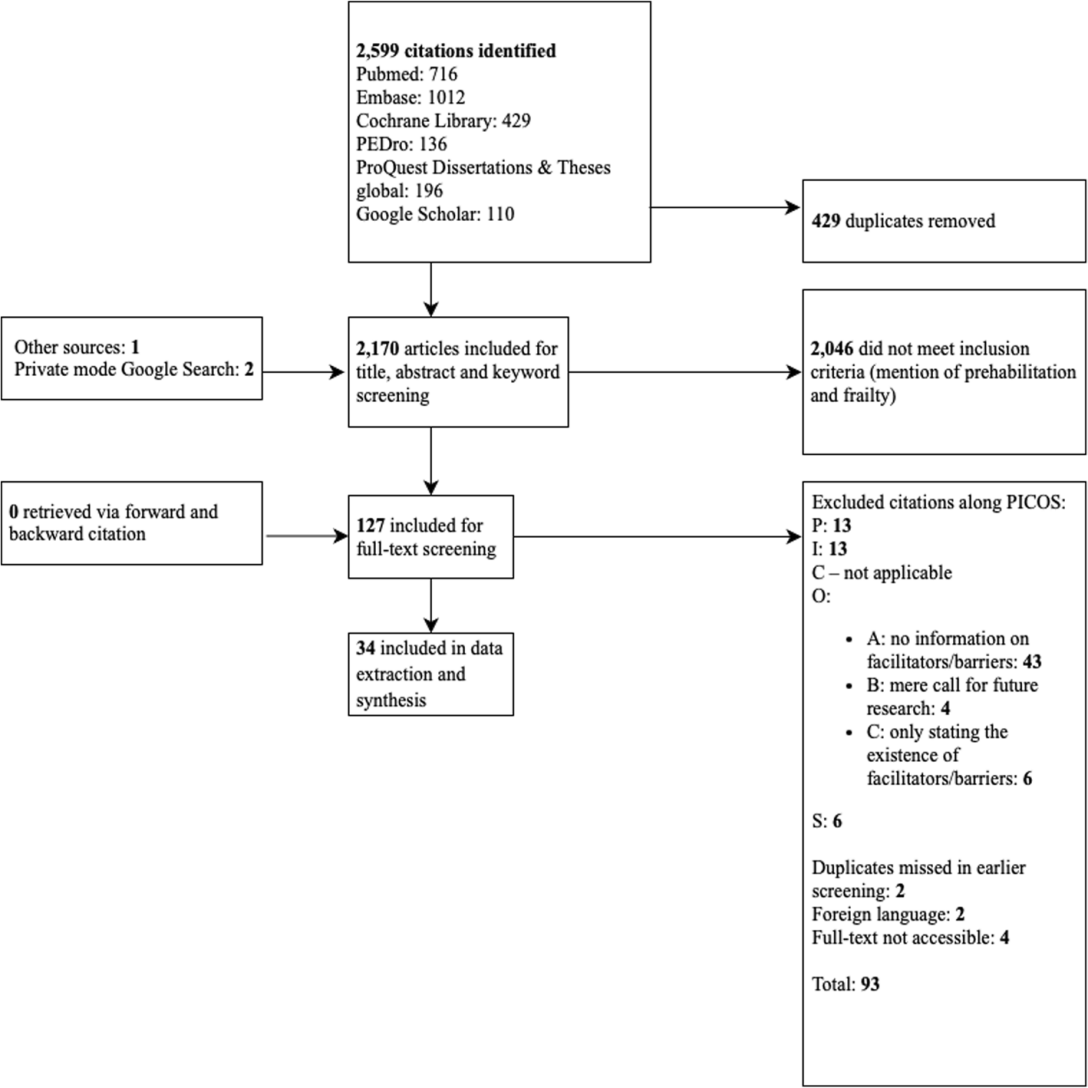
Document flow diagram

Figure 1 shows the screening process, starting with 2,170 unique results from database searches, which were then screened by title and abstract. 127 results met the criteria for full-text screening, of which 34 provided information on facilitators and barriers to the implementation of prehabilitation programmes for frail patients and were thus included in the review [26, 31–63]. Three documents could not be accessed as full texts and were thus excluded [64–66]. The full list of documents excluded after full-text screening can be found in Appendix F.

### Document characteristics

Table 2 provides an overview of the 34 included documents that provide the basis for data synthesis and refinement of the preliminary programme theories to arrive at CMO-configurations for prehabilitation implementation for frail patients. Of the 34 included documents, which were published between 2003 and 2022, four were qualitative, exploratory studies, seven narrative reviews or perspective articles, five randomised clinical trials, eight non-randomised studies, two letters to the editor, as well as two editorials and six systematic reviews. Most of the included publications covered colorectal cancer (*n*=8) or cardiac disease (*n*=5). Eight did not define a disease focus. The prehabilitation interventions ranged from unimodal exercise interventions (*n*=8) to a combined exercise and nutrition intervention (*n*=6) to multimodal (*n*=2) or were not defined (*n*=18).

**Table 2:** Included documents

**References**	**Document type (study design)**	**Location**	**Study Period**	**Sample Size**	**Sample Characteristics**	**Disease Focus**	**Surgery Type**	**Frailty Assessment**	**Description of prehabilitation**
Afilalo [31]	Review article	n.a.	n.a.	n.a.	n.a.	Cardiac disease	Different types	Not defined	Not defined
Agasi-Idenburg et al. [32] & Agasi-Idenburg et al. [33]	Study article (qualitative study)	Netherlands	April 1, 2017 – May 1, 2018	37	15 patients, 13 informal caregivers, 9 healthcare professionals	Colorectal cancer	Colorectal cancer surgery	comorbidity (Chronic Obstructive Pulmonary Disease, Diabetes Mellitus), chemotherapy in the last half year, severe osteoarthrosis, heart failure, or other recent surgery limiting functional capacity	Not defined
Arora et al. [34]	Review article	n.a.	n.a.	n.a.	n.a.	Bladder disease	Radical cystectomy	Not defined	Not defined
Balagué & Arroyo [35]	Editorial	n.a.	n.a.	n.a.	n.a.	Not defined	Not defined	Not defined	Not defined
Bongers et al. [36]	Letter to the editor	n.a.	n.a.	n.a.	n.a.	Colorectal cancer	Colorectal cancer resection	Fried Frailty Index	personalised, supervised, and home-based multimodal programme prescribed by a kinesiologist, a nutritionist, and a psychology-trained nurse; programme started after the baseline visit and continued for 4weeks before surgery
Boreskie et al. [37]	Review article	n.a.	n.a.	n.a.	n.a.	Cardiac disease	Different types	Not defined	Not defined
Bruns et al. [38]	Study article (Non-randomised pilot observational study)	Netherlands	February 2017 – February 2018	14	≥ 70 years who underwent a resection for colorectal cancer	Colorectal cancer	Colorectal cancer surgery	Fried criteria, Clinical Frailty Scale, Short Physical Performance Battery (SPPB), KATZ – Independence of Activities of Daily Living (KATZ-ADL-6 questionnaire)	daily elderly-adapted computer-supported strength training workout (home-based) and two protein-rich meals
Carli et al. [39]	Review article	n.a.	n.a.	n.a.	n.a.	Colorectal cancer	Colorectal cancer surgery	Not defined	Not defined
Durand et al. [40]	Review article	n.a.	n.a.	n.a.	n.a.	Cardiac disease	Different types	Not defined	Not defined
Feng et al. [41]	Study article (nested qualitative study within an RCT)	Ottawa, Canada	Unknown	7 (recruitment ongoing)	patients ≥60 years having elective cancer surgery	Cancer	intraabdominal/intrathoracic surgery	Clinical Frailty Scale	home-based exercise prehabilitation (≥3 weeks of prehabilitation (strength, aerobic, and stretching))
Furyk et al. [42]	Study article (randomised controlled study)	Queensland, Australia	March 2016 – November 2017	5(106 participants eligible for screening)	patient undergoing colorectal surgery for cancer; frail or prefrail; able to attend exercise training in the regional city; and age ≥ 50	Colorectal cancer	Colorectal surgery	Edmonton Frail Scale	4-week supervised exercise program with dietary advice; three 1 h exercise sessions per week on non-consecutive days to increase muscular strength and cardiorespiratory/aerobic function
Gill et al. [43]	Study article (randomised controlled study)	Connecticut, USA	Unknown	94	physically frail, community-living persons, aged 75 years or older	Not defined	Not defined	rapid gait (i.e., walking back and forth over a 3-m course as quickly as possible) and a single chair stand (i.e., standing up from a hard-back chair with arms folded)	home-based physical therapy including progressive balance and conditioning exercises, using Thera-Bands
Gurlit & Gogol [26]	Review article	n.a.	n.a.	n.a.	n.a.	Not defined	Not defined	Not defined	Not defined
Heil et al. [44]	Study article (qualitative study)	Unknown	September 2019 – October 2020	13	5 surgeons, 3 specialised nurses, 3 physical therapists, 2 dieticians	Colorectal cancer	Colorectal cancer surgery	n.a.	prehabilitation = at least aim of improving physical fitness and nutritional status
Hoogeboom et al. [45]	Study article (Single-blind, randomised clinical pilot trial)	Netherlands	July 2007 – November 2008	21	Frail elderly with hip osteoarthritis awaiting total hip replacement	Hip osteoarthritis	Total hip arthroplasty	Clinical Frailty Scale	supervised exercise twice a week (60 minutes each) at an outpatient department of physiotherapy; additionally encouraged to exercise at home; strength and aerobic training, functional physical activities for daily living
Jensen et al. [46]	Review article	n.a.	n.a.	n.a.	n.a.	Bladder disease	Radical cystectomy	Not defined	Not defined
Johanning & Hall [47]	Editorial	n.a.	n.a.	n.a.	n.a.	Not defined	Not defined	Not defined	Not defined
Lin et al. [48]	Study article (Cohort study)	Pennsylvania, USA	July 2018 – July 2019	517	All-comers were included in the analysis because all potential liver transplant candidates received an exercise prescription at their initial PT evaluation	Liver disease	Liver transplantation	Liver Frailty Index	individualised exercise prescription, mainly as home-based exercise workouts; on rare occasions, home health physical therapy or outpatient physical therapy (at a facility close to home) was recommended
McAdams-DeMarco et al. [49]	Study article (Single-arm intervention pilot study)	Maryland, USA	May 2016 – September 2017	24	kidney transplant candidates assessed for frailty	Kidney disease	Kidney transplantation	Fried physical frailty phenotype, Short Physical Performance Battery frailty score	centre-based prehabilitation consisting of weekly physical therapy sessions at an outpatient centre with at-home exercises
Mohamed et al. [50]	Review article	n.a.	n.a.	n.a.	n.a.	Degenerative spine disease	Complex spine surgery	Compares multiple assessments	Not defined
Ng et al. [51]	Review article	n.a.	n.a.	n.a.	n.a.	Not defined	Not defined	Not defined	Not defined
Oosting et al. [52]	Study article (Single-blind pilot randomised controlled trial)	Netherlands	Unknown	30	elective total hip arthroplasty (minimum waiting period of 3 weeks), osteoarthritis as underlying diagnosis for total hip arthroplasty, age older than 65 years; frail patients	End-stage hip osteoarthritis	Total hip arthroplasty	Identification of Seniors at Risk	home-based programme supervised by an experienced physical therapist to train functional activities and walking capacity
Perlmutter et al. [53]	Study article (Observational study)	Ohio, USA	April 2019 – February 2021	32	adult patients to undergo pancreatic resection, surgeries planned for at least 2 weeks after clinic visit	Pancreas disease	Pancreatectomy	Modified Johns Hopkins Frailty Score	daily prehabilitation regimen at home consisting of 100 chair-stands, 30 hand squeezes of a stressball and walking 7,500 steps
Punt et al. [54]	Review/perspective article	n.a.	n.a.	n.a.	n.a.	Not defined	Not defined	Not defined	Not defined
Rumer et al. [55]	Review article	n.a.	n.a.	n.a.	n.a.	Not defined	Not defined	Not defined	Not defined
Shovel & Morkane [56]	Review article	n.a.	n.a.	n.a.	n.a.	Vascular disease	Open and endovascular aortic surgery	Not defined	Not defined
Singer et al. [57] & Singer et al. [58]	Study article (Non-randomised, observational pilot study)	California, USA	December 2015 – November 2017; Start of recruitment in March 2016	15	patients aged ≥50 who were listed or soon to be listed for lung transplantation	Lung disease	Lung transplantation	Short Physical Performance Battery frailty score	home-based combined exercise and nutrition intervention using a commercially available telehealth platform (AidCube)
Wang et al. [59]	Study article (pilot observational study)		Unknown	8	Not defined	Not defined	Not defined	Not defined	4-week prehabilitation programme that includes a blood flow restriction exercise combined with daily consumption of a sports nutrition cocktail; use of a mobile app as a home-based strategyd
Waterland et al. [60]	Letter to the editor	n.a.	n.a.	n.a.	n.a.	Colorectal cancer	Colorectal cancer resection	Fried Frailty Index	personalised, supervised, and home-based multimodal programme prescribed by a kinesiologist, a nutritionist, and a psychology-trained nurse; programme started after the baseline visit and continued for 4 weeks before surgery
Williams et al. [61]	Review article	n.a.	n.a.	n.a.	n.a.	Chronic liver disease	Liver transplantation	Not defined	Not defined
Yau [62] & Yau et al. [63]	Study article (stratified RCT and systematic review)	Hong Kong	July 3, 2019 – December 31, 2020	RCT: 63 (recruitment ongoing), later 153	frail patients (pre-frail to moderately frail) undergoing elective cardiac surgery; non-participants were defined as: inability to regularly attend, or indecisive/refusal to participate	Cardiac disease	Cardiac surgery	Clinical Frailty Scale	preoperative exercise training twice a week to optimise physical and psychosocial fitness at a dedicated room with gymnasium equipment at the Day Surgery Centre

### Main findings

The refined programme theories contain six CMOCs for facilitators and five CMOCs for barriers to the implementation of prehabilitation programmes built (see Tables 3 and 4 and supporting quotations in Appendix G). Of note, some facilitators and barriers can be seen as pairs of antagonists where the presence of one factor may be beneficial, but its absence negatively impacts the outcome.

**Table 3:** Refined programme theories for facilitators to the implementation of prehabilitation for frail patients into routine health care

**CMOC**	**Context**	**+ Mechanism**	**= Outcome**	**References**
1 Well-timed and appropriate information provision	If information about the prehabilitation intervention is provided • in an understandable, “intuitive and user-friendly”([58], p. 7) way, • in a way that emphasises the “value of physical activity and the need to exercise”([33], p. 5) and stresses how prehabilitation helps “perform ADLs [*activities of daily living*]”([31], p. 449) • at an early time point, “since it helps demystify the reason for the intervention”([39], p. 324)	then this enacts • an understanding of the benefits • facilitation of exercise • increased willingness and interest in active participation	resulting in • patient empowerment • increased adherence and motivation • awareness and understanding of one’s own active role in improvement • maintenance of a healthy lifestyle	[31–33, 35, 39, 44, 48, 58]
2 Patient-centred individualisation	If prehabilitation programmes can be • developed with “patient-centered approaches”([37], p. 580) • “easily accessible and take personal preferences, needs and abilities into account”([33], p. 1) • home-based or centre-based, supervised or unsupervised, depending on the patients’ needs, and • “goal-directed, with individualized targets”([51], p. 21)	then this enacts • a feeling of attainability/manageability • patients feeling comfortable with the intervention • stress-free participation • confidence among the patients	resulting in • increased participation • increased adherence and motivation • facilitating implementation in various patient groups	[31–33, 37–39, 41, 43, 44, 46, 48, 51, 52, 54, 56, 58–63]
3 Guidance and (social) support	If the prehabilitation intervention • includes adequate guidance and monitoring by healthcare professionals (digitally and in person), • incorporates goal setting, gamification aspects, and/or rewards, and • integrates the patients’ social environment (family, friends, peers)	then this enacts • enjoyment of the intervention • a feeling of accountability and security with the intervention • emotional and psychological well-being	resulting in • increased adherence • increased participation • self-affirmation by the patients	[33, 36, 38, 44, 45, 48, 53, 55–57, 59, 61]
4 Integration into and adaption of the setting	If prehabilitation programmes can be • “integrated in the perioperative trajectory and performed in the patient’s preexistent living context”([36], p. 896), • “administered within the scope of multidisciplinary collaboration and as an integrated concept”([26], p. 112), and • diffused by an ambassador	then this enacts access to and acceptance of the programme by patients and providers alike	resulting in • more opportunities and motivation to implement prehabilitation interventions into a given setting • increased participation • increased adherence	[26, 33, 34, 36, 39, 43, 44, 61]
5 Multidisciplinary team approach	If, in a multidisciplinary team, • prehabilitation is “understood as an appeal to cooperation between all professions involved”([26], p. 112), • leadership and responsibilities are clear, and • a “shift in the current health care paradigm”([46], p. 6) can be achieved	then this enacts • a new understanding of a common purpose, • an understanding of roles, • mutual respect and support	resulting in • more integrated care, • more cooperation and teamwork • maximised benefit for the patient	[26, 39, 44, 46, 51, 56, 62]
6 Clear patient pathway	If there are • specific and early entry points, • clear referral guidelines, and • possibilities “to lengthen the time interval between operation indication and surgery”([44], p. 11)	then this enacts • smooth referral of patients between disciplines, • shared accountability, • sufficient time for the intervention	resulting in • maximisation of the benefits of prehabilitation, • care integration, • optimal use of resources	[26, 39, 44, 48]

**Table 4:** Refined Programme theories for barriers to the implementation of prehabilitation for frail patients into routine health care

**CMOC**	**Context**	**+ Mechanism**	**= Outcome**	**References**
1 Overwhelming and/or inadequate information provision	If information is provided • at an inappropriate time when patients are “minimally able to process further information”([42], p. 3) • in a non-engaging, imprecise manner, and • does not address patients’ incorrect conceptions of health behaviour	then this enacts • overwhelming of the patients, • no engagement and understanding for the benefit of the programme by the patient, and • continuance of detrimental behaviour	Resulting in • no motivation/will to participate, • no awareness of own role in improving pre-surgery, and • difficulties in adherence	[31, 33, 38, 42, 44, 50, 52, 56]
2 Lack of multi-modality and/or adaptability	If the prehabilitation programme • is a “one-size-fits-all intervention”([38], p. 13), • is not adaptable to the individual capabilities, needs and mobility of the patient, e.g., if there is “inflexibility of ‘prescribed’ prehabilitation”([44], p. 11), and • is not adapted to the local setting	then this enacts • excessive demand on the patients (feeling overwhelmed), • extra stresses, • dissatisfaction with the intervention	resulting in • low compliance or drop out, • inability to participate in or even access the intervention (e.g., due to long distances), • exclusion of patient groups	[31, 38, 40–45, 48, 49, 52, 54, 56, 59, 61]
3 Fragmentation and misalignment of providers	If providers • do not endorse the prehabilitation intervention equally, • “are unaware of (the importance) of prehabilitation programs”([44], p. 4), and • if parts of the patient pathway take precedence over others	then this • enacts “miscommunication and misaligned goals among the healthcare team and lack of commitment among the patients”([51], p. 21), • enacts a lack of common purpose, and • disturbs the referral of patients	resulting in • difficulties in implementation, • difficulties in maximising the benefits of the intervention, and • lack of care integration • tension between different professions along the care pathway	[33, 34, 44, 47, 48, 50, 51]
4 Resource constraints	If the “clinical demand could outstrip existing resources, both human and financial”([47], p. 1) and there is a lack of reimbursement	then this enacts • lack of acceptance for the implementation, and • variability in content of prehabilitation provided	resulting in • exhaustion, • lack of sustainability, and • suboptimal and limited prehabilitation provision	[33, 34, 37, 40, 44, 47, 48, 50, 51, 56, 61, 62]
5 Lack of (social) support	If there is a “lack of physician support, attributed to a lack of conviction regarding the benefit of prehabilitation”([51], p. 21) and if patients feel like a burden to their family and friends, especially due to transportation needs	then this enacts • lack of focus on the intervention, • emotional/psychological stress, and • uncertainty about the importance of the intervention	resulting in • difficulties in compliance/adherence, • limited success, and • non-participation/drop-out of patients	[42, 51]

As part of the refinement process, a CMOC on “Guidance and (social) support” was added in the programme theory on facilitators. This theme had emerged as a unique domain which might greatly affect the feasibility of prehabilitation among frail patients. The themes covered by the refined CMOCs are described in detail in the following paragraphs.

### Information provision

Information provision (when, how and by whom) impacts whether patients feel overwhelmed, can process the information, and understand their own role in the intervention. Although it is recommended to approach prospective patients as early as possible to allow for sufficient time for prehabilitation, it is often overwhelming for patients to process information after receiving their diagnosis and need for surgery, leading to limited willingness to participate [33, 42]. Information should suit the patients’ prior knowledge about the components of prehabilitation, conveyed in an understandable way and emphasise the importance of prehabilitation for activities of daily living [31, 44]. Comprehending the intervention and its benefits, especially for their independence in daily activities after surgery, leads to the patients’ understanding of their own role in affecting the outcome of surgery. This improves compliance and increases motivation and adherence to the prehabilitation programme [33, 35].

### Patient-centredness and programme adaptability

Adaptability is one of the most important features for successful prehabilitation implementation, especially for frail individuals, who are generally more limited in their mobility and tasks they can do independently. If a prehabilitation programme is a “one-size-fits-all intervention” ([38], p. 13) and not adaptable to the individual capabilities, needs and mobility, this can lead to excessive demand, leaving the frail patients feeling overwhelmed [44, 48]. Individualising exercises, nutrition and psychological advice and adapting them to the lifestyles and degree of frailty is important to ensure compliance and a feeling of comfort and attainability for the patients.

The possibility to complete the intervention at home is helpful for frail patients because of their limited mobility: transportation is a significant barrier for participation. Dependency on others to be transported to participate in sessions makes patients feel like a burden and prevents regular attendance or any participation [31, 42, 45, 48, 49, 52, 54, 56, 59]. Despite less supervision, support by a health care professional and equipment being available in the patients’ homes, home programmes can significantly increase accessibility and is often preferred by patients [31, 37, 41, 43, 58]. Video-conferencing and digital tools like wearable fitness trackers or health apps are helpful in remote monitoring of the patients [38, 43, 56].

### Guidance and (social) support

Frail patients need significant support, both by professionals and their social networks, to successfully participate in prehabilitation programmes. Physician support is needed to reduce patients’ uncertainty about the importance of the intervention. Professionals should monitor the patient’s activities, set goals and rewards together with the patients to make them feel directed within the prehabilitation programme [36, 44, 48, 53, 56, 59, 61].

Because of their limited independence, frail patients often strongly rely on their social network, especially for transportation, making support by friends and family important [42]. Without social support, participating in a prehabilitation programme can be emotionally and psychologically stressful, leading to difficulties in adherence and compliance, limited success in and benefit from the programme, or even increased drop-out or non-participation rates.

### Integration into and adaption of the setting

For the prehabilitation programme to be successful across different settings, it needs be integrated into the local setting, which depends on factors such as contextual readiness, expressed as leadership support, but also flexibility in hospital and surgery culture as well as available resources [34]. Diffusion and promotion by an ambassador, who is well respected and trusted within the local setting, can increase acceptance [26, 44]. Low cost prehabilitation programmes can facilitate the uptake in settings where financial resources are scarce [33, 61]. For successful integration into the perioperative patient pathway, changes in the organisation processes will be needed to be adopted by all involved stakeholders [26, 44].

### Resources

Multimodal prehabilitation is a resource-intensive intervention [34, 50, 51, 56]. The resource intensity is determined by the degree of support a patient needs to complete the program and if it is a home-based or centre-based intervention. The availability of human and financial resources can vary by location as well as the time frame available for the intervention prior to surgery depending on the urgency of the diagnosis and waitlists [37, 62]. A difficulty in prehabilitation implementation is that it is an intervention, which does not show immediate effects, however needs significant funding up front [44], making investment difficult to obtain. If prehabilitation is implemented despite resource constraints, it can put an additional strain on personnel and the quality of care provided [61].

### Multidisciplinary team approach

The adoption of prehabilitation programmes into the perioperative trajectory depends on a multidisciplinary and interprofessional team approach, because such an intervention among frail and multimorbid patients requires a holistic approach to adequately address their needs [51]. Only if all involved healthcare professionals have the same understanding of the way prehabilitation should be integrated into the health care setting and the intervention is perceived as valuable by all players, can patients benefit from well-integrated, multimodal care that produces the best outcomes [26]. Patient selection as well as the timing and individual design of the intervention require discussions and cooperation between different providers so that frail patients get the maximum benefit from prehabilitation. If there are no predefined guidelines for prehabilitation, e.g., including a minimum duration, the referral to the intervention could be disturbed and tensions among professionals along the care pathway can arise, sabotaging prehabilitation goals.

### Clear patient pathway

A clear patient pathway is facilitated if there are specific and early entry points that follow clear and accepted referral guidelines. It is important that not only the selection criteria for patients to participate in prehabilitation are clear, but that patients enter the prehabilitation programme as early as possible to allow for sufficient time for the intervention before the date of surgery [26, 39, 48]. Ideally, the patient pathway should allow for the flexibility regarding the duration between diagnosis including indication for surgery and the procedure [44]. Clear guidelines, and at the same time, a degree of flexibility, enact a smooth referral between different healthcare professionals, allowing for shared accountability for the success of the intervention. Ultimately, this helps to optimally use existing resources and to maximise the benefits of the prehabilitation programme.

### Conflict of interest & funding of the included documents

Information on funding and conflict of interest can be found in Appendix H. Six of the included documents declared that there was no funding and no conflict of interest to report [26, 38, 51, 53, 56, 61]. Six documents did not report their funding and potential conflict of interest and should be considered with caution [32, 35, 39, 41, 62, 63]. Eleven documents, which either did not report a conflict of interest or reported no conflict of interest, received research grants or sponsoring by different national research institutions (non-profit funding), whereas seven documents reported a combination of “none” and “not reported” for conflict of interest and funding [21, 36, 40, 46, 50, 59, 60]. Four other documents declared a conflict of interest, including a CEO position in a firm providing services used in the study [58], a consulting position and intellectual property ownership [47], and financial contributions by medical firms unrelated to the research [34, 37]. The former can be considered problematic as this direct involvement can compromise the evaluation of the study, whereas the readers should be aware of the two latter declarations, however, are not of the same degree of conflict as the first.

### Relevance and rigour of the included documents

Out of the 34 included documents, five were classified as being of less rigour because they express personal opinions (letters to the editor, editorials) and the quality of these contributions vary with expertise of the authors. Eight documents provide context-specific insights with high rigour that were obtained through observations and interviews, whereas the remaining 21 documents provided more generalised information that was obtained through reviews.

In terms of relevance, all documents were considered relevant as they added to the analysis of barriers and facilitators to the implementation of prehabilitation for frail patients. However, the documents had varying degrees of relevance based on the type of information they provided. Ten documents were conducted in a controlled experimental setting, not reflecting real-world experiences, which made them less relevant than five other documents that brought insights from real-life settings through observation and interviews. 17 publications provided generalised information that can be considered more relevant to answer questions regarding implementation than those from controlled settings, however, cannot provide the same relevance as context-specific experiences. An overview of judgements of relevance and rigour for each publication can be found in Appendix I.

## Discussion

### Summary of findings

This realist review provides insights on facilitator and barriers for the implementation of prehabilitation programmes for frail patients who are planning to undergo elective surgery. Six facilitating factors and five barriers were identified, which can be seen as pairs of antagonists whose presence is beneficial, while the absence has negative impact. Most facilitating and hindering factors for the implementation of prehabilitation programmes apply to both frail and non-frail patient groups. For the successful implementation of prehabilitation programmes, the organisational readiness must be given. This includes resources, such as reimbursement, staff, premises, as well as the willingness to implement new and clear referral guidelines and to integrate prehabilitation into the patient pathway. This also necessitates a common understanding and purpose among the health care providers who are part of the multimodal prehabilitation team. Additionally, health care professionals should adapt their communication to fit frail patients. The timing and manner of information provision is also essential to effectively reach and not overwhelm patients and is complicated by the time available until surgery.

Frail patients are limited in their independence, physically, mentally, or both, and require special guidance and support to be able to complete a prehabilitation programme. Support by family and friends plays a vital role, because prehabilitation can be psychologically and physically stressful. Central to the individualisation for frail people is the possibility to participate in a home-based intervention as transportation to attend centre-based programmes is one of the most significant barriers to implementing prehabilitation for frail people. Home-based prehabilitation, however, still needs to provide sufficient support and should ideally be multimodal. In addition, home-based prehabilitation can also be limiting when space is restricted or if monitoring is not feasible [54].

### Comparison with existing literature

The findings from this realist review are in line with factors that are frequently named in theoretical implementation science frameworks. Wisdom et al. [67] reviewed 20 theoretical adoption frameworks for the implementation process of complex interventions and found 28 factors on five levels of adoption: Socio-political and External Influence, Organisation Characteristics, Innovation Characteristics, Staff/Individual Characteristics, and Client Characteristics [67]. The facilitators and barriers identified in this realist review fit within these five levels of adoption and are also comparable to factors within the levels of adoption. For example, the factor “Leadership and Champion of Innovation”, part of “Organization Characteristics”, claims that organisational leadership when promoting an innovation is an essential component for successful pre-adoption and adoption [67]. The importance of an ambassador is also recognised in this review.

Findings related to frail patients’ perceptions of and problems with prehabilitation in this review were primarily gained from the qualitative studies by Agasi-Idenburg et al. [33] and Heil et al. [44]. A commonly used framework for qualitative studies on health behaviour change is the Theoretical Domains Framework (TDF), which contains 14 domains that guide the assessment of behaviour change as a result of an intervention [68]. This framework can be helpful to analyse findings from stakeholder interviews. Barnes et al. 2023 conducted a qualitative study that was published after our date of last search [69] using TDF to identify barriers and facilitators to participation in exercise prehabilitation before cancer surgery for older adults with frailty. Their study was nested into the RCT by McIsaac et al. 2020 [14] and found that home-based prehabilitation programs are manageable for frail patients with adequate support and can lead to self-perceived health benefits [69]. Similar to our study, Barnes et al. 2023 found that the need for individualisation, adoptability and variety is a key determinant for the success of prehabilitation interventions among frail patients [69].

### Strengths and limitations

The strengths of this realist review include that it was registered prior to conducting the research. Furthermore, by drawing on quantitative, qualitative, and review data, this realist review combines multiple perspectives and experiences to create program theories that can be applied to many situations of prehabilitation implementation. The search strategy produced a broad range of publications and there was no restriction on the publication date so that early as well as very recent studies on prehabilitation were included. Different geographical and health care settings and a variety of prehabilitation approaches were included, so that many different factors impacting implementation are covered in this review.

Limitations posed by the review methods include that, although the search approach aimed to be as comprehensive as possible, literature adding to the issue might have been missed. Only texts in English and German were included, which could have introduced a language bias. Additionally, three full texts were not accessible and subsequently had to be excluded [64–66]. Due to limited resources, screening was performed independently and in duplicate only in a subset and (following high agreement) continued by one person. Also, data was only extracted by one researcher, which might have introduced errors. Lastly, the assessment of relevance and rigour is subjective to a certain degree, leaving room for debate to what extent each of the included articles provide quality information on facilitators and barriers of the implementation of prehabilitation for frail patients.

The included literature itself also poses some limitations. For one thing, the included articles vary methodologically, and multiple definitions of frailty are used, which can lead to a different understanding of the patients’ limitations, and thus, a different ability to complete a prehabilitation programme. Additionally, the content and intensity of the prehabilitation interventions varied or were not defined, which complicates the interpretation and generisability of results.

## Conclusions

We identified several context-mechanism-outcome-configurations for facilitators and barriers to the implementation of prehabilitation programmes prior to elective surgery for frail patients. The resulting programme theories show that, when designing the prehabilitation programme, it is key that the intervention is individualised to fit the capacities and needs of the frail patient. This should be done in cooperation with the patients and their social environment. Furthermore, adequate information provision by health care professionals leads to an understanding of the importance of the intervention and the patients’ role in improving their outcomes, which can be enforced by regular communication with the patient and family. When introducing the prehabilitation programme into routine care, change management activities are required to transform the care pathway. Organisational readiness must include resources, commitment and endorsement by the multidisciplinary team, a clear referral system and clear distribution of responsibilities along the patient pathway. While it is important to learn from the successes and failures of other prehabilitation programmes, the programme must be adapted to the local setting, e.g. after a pilot phase with thorough evaluation.

### Supplementary Information

**Additional file 1.**

## Data Availability

All extracted data is presented in the tables and appendices.

## Abbreviations

ADLs: activities of daily living
CEO: Chief Executive Officer
CMO: context-mechanism-outcome
CMOC: context-mechanism-outcome configurations
PEDro: Physiotherapy Evidence Database
PICOSH: Population, Intervention, Comparison/Control, Outcome, Study design, Healthcare context
RCT: randomised controlled trial
TDF: Theoretical Domains Framework
