# Supplementary material for: Facilitators and barriers to the implementation of prehabilitation for frail patients into routine health care: a realist review
Source: BMC Health Serv Res. 2024 Feb 13;24:192. doi: 10.1186/s12913-024-10665-1 (PMC10863196; doi:10.1186/s12913-024-10665-1)
Supplement: Supplementary file 1 — Additional file 1. [file 12913_2024_10665_MOESM1_ESM.docx]

# Supplementary materials

## Appendix A: RAMESES II Reporting Checklist

| **Reporting item** | | **Description of item** | **Reported on page(s)** |
| --- | --- | --- | --- |
| **Title** | | | |
| 1 |  | In the title, identify the document as a realist synthesis or review | 1 |
| **Abstract** | | | |
| 2 |  | While acknowledging publication requirements and house style, abstracts should ideally contain brief details of: the study’s background, review question or objectives; search strategy; methods of selection, appraisal, analysis and synthesis of sources; main results; and implications for practice | 2 |
| **Introduction** | | | |
| 3 | Rationale for review | Explain why the review is needed and what it is likely to contribute to existing understanding of the topic area | 4-5 |
| 4 | Objectives and focus of review | State the objective(s) of the review and/or the review question(s). Define and provide a rationale for the focus of the review | 5 |
| **Methods** | | | |
| 5 | Changes in the review process | Any changes made to the review process that was initially planned should be briefly described and justified | 5 |
| 6 | Rationale for using realist synthesis | Explain why realist synthesis was considered the most appropriate method to use | 5-6 |
| 7 | Scoping the literature | Describe and justify the initial process of exploratory scoping of the literature | 6 |
| 8 | Searching processes | While considering specific requirements of the journal or other publication outlet, state and provide a rationale for how the iterative searching was done. Provide details on all of the sources accessed for information in the review. Where searching in electronic databases has taken place, the details should include, for example, name of database, search terms, dates of coverage and date last searched. If individuals familiar with the relevant literature and/or topic area were contacted, indicate how they were identified and selected | 6-7 |
| 9 | Selection and appraisal of documents | Explain how judgements were made about including and excluding data from documents, and justify these | 7-8 |
| 10 | Data extraction | Describe and explain which data or information were extracted from the included documents and justify this selection | 8-9 |
| 11 | Analysis and synthesis processes | Describe the analysis and synthesis processes in detail. This section should include information on the constructs analysed and describe the analytic process | 9 |
| **Results** | | | |
| 12 | Document flow diagram | Provide details on the number of documents assessed for eligibility and included in the review, with reasons for exclusion at each stage, as well as an indication of their source of origin (e.g. from searching databases, reference lists and so on). You may consider using the example templates (which are likely to need modification to suit the data) that are provided | 11 |
| 13 | Document characteristics | Provide information on the characteristics of the documents included in the review | 11-12 |
| 14 | Main findings | Present the key findings with a specific focus on theory building and testing | 13-17 |
| **Discussion** | | | |
| 15 | Summary of findings | Summarise the main findings, taking into account the reviews objective(s), research question(s), focus and intended audience(s) | 17-18 |
| 16 | Strengths, limitations and future research directions | Discuss both the strengths of the review and its limitations. These should include (but need not be restricted to) (a) consideration of all the steps in the review process and (b) comment on the overall strength of evidence supporting the explanatory insights which emerged The limitations identified may point to areas where further work is needed | 19-20 |
| 17 | Comparison with existing literature | Where applicable, compare and contrast the reviews findings with the existing literature (e.g. other reviews) on the same topic | 18-19 |
| 18 | Conclusion and recommendations | List the main implications of the findings and place these in the context of other relevant literature. If appropriate, offer recommendations for policy and practice | 20-21 |
| 19 | Funding | Provide details of funding source (if any) for the review, the role played by the funder (if any) and any conflicts of interests of the reviewers | 22-23 |

## Appendix B: PRISMA 2020 Reporting Checklist

| **Section and Topic** | **Item #** | **Checklist item** | **Location where item is reported** |
| --- | --- | --- | --- |
| **TITLE** | | |  |
| Title | 1 | Identify the report as a systematic review. | Not applicable (realist review); instead identified as realist review on p. 1 |
| **ABSTRACT** | | |  |
| Abstract | 2 | See the PRISMA 2020 for Abstracts checklist. | 2 |
| **INTRODUCTION** | | |  |
| Rationale | 3 | Describe the rationale for the review in the context of existing knowledge. | 4-5 |
| Objectives | 4 | Provide an explicit statement of the objective(s) or question(s) the review addresses. | 5 |
| **METHODS** | | |  |
| Eligibility criteria | 5 | Specify the inclusion and exclusion criteria for the review and how studies were grouped for the syntheses. | 7-8, Table 1 |
| Information sources | 6 | Specify all databases, registers, websites, organisations, reference lists and other sources searched or consulted to identify studies. Specify the date when each source was last searched or consulted. | 6-7 |
| Search strategy | 7 | Present the full search strategies for all databases, registers and websites, including any filters and limits used. | Appendix C |
| Selection process | 8 | Specify the methods used to decide whether a study met the inclusion criteria of the review, including how many reviewers screened each record and each report retrieved, whether they worked independently, and if applicable, details of automation tools used in the process. | 7-8 |
| Data collection process | 9 | Specify the methods used to collect data from reports, including how many reviewers collected data from each report, whether they worked independently, any processes for obtaining or confirming data from study investigators, and if applicable, details of automation tools used in the process. | 8 |
| Data items | 10a | List and define all outcomes for which data were sought. Specify whether all results that were compatible with each outcome domain in each study were sought (e.g. for all measures, time points, analyses), and if not, the methods used to decide which results to collect. | 8-9 |
|  | 10b | List and define all other variables for which data were sought (e.g. participant and intervention characteristics, funding sources). Describe any assumptions made about any missing or unclear information. | 8-9 |
| Study risk of bias assessment | 11 | Specify the methods used to assess risk of bias in the included studies, including details of the tool(s) used, how many reviewers assessed each study and whether they worked independently, and if applicable, details of automation tools used in the process. | Not applicable (realist review); instead, relevance and rigor were assessed (p. 9-10) |
| Effect measures | 12 | Specify for each outcome the effect measure(s) (e.g. risk ratio, mean difference) used in the synthesis or presentation of results. | Not applicable (realist review) |
| Synthesis methods | 13a | Describe the processes used to decide which studies were eligible for each synthesis (e.g. tabulating the study intervention characteristics and comparing against the planned groups for each synthesis (item #5)). | 9 |
|  | 13b | Describe any methods required to prepare the data for presentation or synthesis, such as handling of missing summary statistics, or data conversions. | Not applicable (realist review) |
|  | 13c | Describe any methods used to tabulate or visually display results of individual studies and syntheses. | 9 |
|  | 13d | Describe any methods used to synthesize results and provide a rationale for the choice(s). If meta-analysis was performed, describe the model(s), method(s) to identify the presence and extent of statistical heterogeneity, and software package(s) used. | 9 |
|  | 13e | Describe any methods used to explore possible causes of heterogeneity among study results (e.g. subgroup analysis, meta-regression). | Not applicable (realist review) |
|  | 13f | Describe any sensitivity analyses conducted to assess robustness of the synthesized results. | Not applicable (realist review) |
| Reporting bias assessment | 14 | Describe any methods used to assess risk of bias due to missing results in a synthesis (arising from reporting biases). | Not applicable (realist review) |
| Certainty assessment | 15 | Describe any methods used to assess certainty (or confidence) in the body of evidence for an outcome. | Not applicable (realist review) |
| **RESULTS** | | |  |
| Study selection | 16a | Describe the results of the search and selection process, from the number of records identified in the search to the number of studies included in the review, ideally using a flow diagram. | 10-11, Figure 1 |
|  | 16b | Cite studies that might appear to meet the inclusion criteria, but which were excluded, and explain why they were excluded. | 11 |
| Study characteristics | 17 | Cite each included study and present its characteristics. | 11-12 |
| Risk of bias in studies | 18 | Present assessments of risk of bias for each included study. | Not applicable (realist review); instead, relevance and rigor were assessed (results on p. 13) |
| Results of individual studies | 19 | For all outcomes, present, for each study: (a) summary statistics for each group (where appropriate) and (b) an effect estimate and its precision (e.g. confidence/credible interval), ideally using structured tables or plots. | Table 3, Table 4 |
| Results of syntheses | 20a | For each synthesis, briefly summarise the characteristics and risk of bias among contributing studies. | Not applicable (realist review) |
|  | 20b | Present results of all statistical syntheses conducted. If meta-analysis was done, present for each the summary estimate and its precision (e.g. confidence/credible interval) and measures of statistical heterogeneity. If comparing groups, describe the direction of the effect. | Not applicable (realist review) |
|  | 20c | Present results of all investigations of possible causes of heterogeneity among study results. | Not applicable (realist review) |
|  | 20d | Present results of all sensitivity analyses conducted to assess the robustness of the synthesized results. | Not applicable (realist review) |
| Reporting biases | 21 | Present assessments of risk of bias due to missing results (arising from reporting biases) for each synthesis assessed. | Not applicable (realist review) |
| Certainty of evidence | 22 | Present assessments of certainty (or confidence) in the body of evidence for each outcome assessed. | Not applicable (realist review) |
| **DISCUSSION** | | |  |
| Discussion | 23a | Provide a general interpretation of the results in the context of other evidence. | 17-19 |
|  | 23b | Discuss any limitations of the evidence included in the review. | 20 |
|  | 23c | Discuss any limitations of the review processes used. | 20 |
|  | 23d | Discuss implications of the results for practice, policy, and future research. | 20-21 |
| **OTHER INFORMATION** | | |  |
| Registration and protocol | 24a | Provide registration information for the review, including register name and registration number, or state that the review was not registered. | 5 |
|  | 24b | Indicate where the review protocol can be accessed, or state that a protocol was not prepared. | 5 |
|  | 24c | Describe and explain any amendments to information provided at registration or in the protocol. | 5 |
| Support | 25 | Describe sources of financial or non-financial support for the review, and the role of the funders or sponsors in the review. | 23 |
| Competing interests | 26 | Declare any competing interests of review authors. | 22-23 |
| Availability of data, code and other materials | 27 | Report which of the following are publicly available and where they can be found: template data collection forms; data extracted from included studies; data used for all analyses; analytic code; any other materials used in the review. | 22 |

## Appendix C: Database Searches

Database searches run on June 7, 2022

**Medline via Pubmed:**

("prehab*"[TIAB] OR "pre hab*"[TIAB] OR "preoperative exercise"[MeSH Terms] OR "Preoperative Care"[MeSH] OR "preoperative exercise"[TIAB] OR "presurgical exercise"[TIAB] OR "preoperative training"[TIAB] OR "presurgical training"[TIAB] OR "preoperative physical therapy"[TIAB] OR "presurgical physical therapy"[TIAB] OR "preoperative occupational therapy"[TIAB] OR "presurgical occupational therapy"[TIAB] OR "preoperative physiotherapy"[TIAB] OR "presurgical physiotherapy" [TIAB] OR "preoperative physical activity"[TIAB] OR "presurgical physical activity"[TIAB] OR "preoperative fitness"[TIAB] OR "presurgical fitness"[TIAB] OR "preoperative conditioning"[TIAB] OR "presurgical conditioning"[TIAB] OR "preconditioning"[TIAB] OR "preoperative habilitation"[TIAB] OR "presurgical habilitation"[TIAB] OR "preoperative rehabilitation"[TIAB] OR "presurgical rehabilitation"[TIAB] OR "preoperative education"[tiab] OR "presurgical education "[TIAB] OR "Anesthesiology/methods"[MAJR]) AND ("frail*"[TIAB] OR "frailty"[MeSH Terms] OR "frail elderly"[MeSH Terms] OR "health services for the aged"[MeSH Terms] OR (("aged"[TIAB] OR "elder*"[TIAB] OR "Geriatrics"[MeSH] OR "geriatric*"[TIAB] OR "gerontol*"[TIAB] OR "septuagenarian"[TIAB] OR "octogenarian"[TIAB] OR "nonagenarian"[TIAB]) AND ("comorbid*"[TIAB] OR "comorbidity"[MeSH] OR "vulnerab*"[TIAB] OR "multimorbid*"[TIAB] OR "high risk"[TIAB])))

**Embase:**

1. (prehab* or pre hab* or (preoperative adj3 exercise) or (preoperative adj3 care) or (preoperative adj3 habilitation) or (presurgical adj3 habilitation) or (preoperative adj3 exercise) or (presurgical adj3 exercise) or (preoperative adj3 training) or (presurgical adj3 training) or (preoperative adj3 physical adj3 therapy) or (presurgical adj3 physical adj3 therapy) or (preoperative adj3 occupational adj3 therapy) or (presurgical adj3 occupational adj3 therapy) or (preoperative adj3 physiotherapy) or (preoperative adj3 physical adj3 activity) or (presurgical adj3 physical adj3 activity) or (preoperative adj3 fitness) or (presurgical adj3 fitness) or (preoperative adj3 conditioning) or (presurgical adj3 conditioning) or preconditioning or (preoperative adj3 rehabilitation) or (presurgical adj3 rehabilitation) or (preoperative adj3 education) or (presurgical adj3 education)).ab,kw,ti.

2. exp preoperative exercise/

3. exp preoperative care/

4. exp frailty/

5. exp frail elderly/

6. exp elderly care/

7. frail*.ab,kw,ti.

8. 1 or 2 or 3

9. 4 or 5 or 6 or 7

10. aged.ab,kw,ti.

11. elder*.ab,kw,ti.

12. exp geriatrics/

13. geriatric*.ab,kw,ti.

14. Gerontol*.ab,kw,ti.

15. septuagenarian.ab,kw,ti.

16. octogenarian.ab,kw,ti.

17. nonagenarian.ab,kw,ti.

18. 10 or 11 or 12 or 13 or 14 or 15 or 16 or 17

19. comorbid*.ab,kw,ti.

20. exp comorbidity/

21. multimorbid*.ab,kw,ti.

22. (high adj3 risk).ab,kw,ti.

23. vulnerab*.ab,kw,ti.

24. 19 or 20 or 21 or 22 or 23

25. 18 and 24

26. 9 or 25

27. 8 and 26

**Cochrane Library:**

#1 prehab* 502

#2 pre hab* 6105

#3 MeSH descriptor: [Preoperative Exercise] explode all trees 38

#4 MeSH descriptor: [Preoperative Exercise] explode all trees 38

#5 MeSH descriptor: [Preoperative Care] explode all trees 6133

#6 preoperative exercise 1379

#7 preoperative habilitation 22

#8 presurgical habilitation 3

#9 presurgical exercise 86

#10 preoperative training 1598

#11 presurgical training 88

#12 preoperative physical therapy 1323

#13 presurgical physical therapy 81

#14 preoperative occupational therapy 57

#15 presurgical occupational therapy 10

#16 preoperative physiotherapy 595

#17 preoperative physical activity 707

#18 presurgical physical activity 63

#19 preoperative fitness 262

#20 presurgical fitness 28

#21 preoperative conditioning 142

#22 presurgical conditioning 18

#23 preconditioning 2002

#24 preoperative rehabilitation 1600

#25 presurgical rehabilitation 72

#26 preoperative education 1512

#27 presurgical education 85

#28 #1 OR #2 OR #3 OR #4 OR #5 OR #6 OR #9 OR #10 OR #11 OR #12 OR #13 OR #14 OR #15 OR #16 OR #17 OR #18 OR #19 OR #20 OR #21 OR #22 OR #23 OR #24 OR #25 OR #26 OR #27 18997

#29 frail* 4779

#30 MeSH descriptor: [Frailty] explode all trees 275

#31 MeSH descriptor: [Frail Elderly] explode all trees 783

#32 MeSH descriptor: [Health Services for the Aged] explode all trees 462

#33 MeSH descriptor: [Geriatrics] explode all trees 213

#34 gerontol* 4310

#35 aged 550101

#36 geriatric 12307

#37 septuagenarian 8

#38 octogenarian 38

#39 nonagenarian 8

#40 #29 OR #30 OR #31 OR #32 5152

#41 #33 OR #34 OR #35 #36 OR #37 OR #38 OR #39 12321

#42 comorbid* 29403

#43 vulnerab* 8888

#44 MeSH descriptor: [Comorbidity] explode all trees 3828

#45 multimorbid* 678

#46 high risk 104022

#47 #42 OR #43 OR #44 OR #45 OR #46 133605

#48 #41 AND #47 1969

#49 #48 OR #40 6731

#50 #28 AND #49 427

**PEDro:**

Prehab*

**Google Scholar:**

prehab* OR "preoperative exercise" OR "preoperative care" OR preoperative exercise" OR presurgical exercise" OR "preoperative training" OR "presurgical training" OR "preoperative physical therapy" OR "presurgical physical therapy" OR "preoperative occupation therapy" OR "presurgical occupational therapy" OR "preoperative physiotherapy" OR "presurgical physiotherapy" OR "preoperative physical activity" OR "presurgical physical activity" OR "preoperative fitness" OR "presurgical fitness" OR "preoperative conditioning" OR "presurgical conditioning" OR "preconditioning" OR "preoperative habilitation" OR "presurgical habilitation" OR "preoperative rehabilitation" OR "presurgical rehabilitation" OR "preoperative education" OR "presurgical education" AND "frail*" OR health services for the aged"

**ProQuest Dissertations & Theses Global:**

Prehab*

## Appendix D: Preliminary Programme Theories for Facilitators to the Implementation of Prehabilitation into Routine Health Care

| **CMOC** | **Context** | **+ Mechanism** | **= Outcome** | **References** |
| --- | --- | --- | --- | --- |
| 1  Well-timed and appropriate information provision | If information about the prehabilitation intervention is provided   - at a convenient time “in relation to the timing of surgery” ( (1), p. 9), - in an engaging and clear format, - in “manageable ‘chunks’” ( (2), p. 142), and - adapted to the patient’s existing knowledge about the (pre-)habilitation programmes | Then this enacts   - comprehension and information processing by the patients - stimulation of patients’ interest in active participation | Resulting in   - increased adherence and motivation - an understanding of one’s own active role in improvement | (1-4) |
| 2  Patient-centred individualization | If prehabilitation programmes can be   - “developed with a person-centred approach, - considering individual needs and preferences” ( (5), p. 2), and - taking into account their mobility radius in terms of access to health care services | Then this enacts   - a feeling of attainability/manageability - confidence among the patients - no extra stresses caused | Resulting in   - easy participation - increased adherence - facilitating implementation in various patient groups | (2) (5-8) |
| 3  Integration into and adaption of the setting | If prehabilitation programmes can be   - tailored to “best suit the local context” ( (5), p. 2), and - adapted to existing infrastructure, e.g., the primary point(s) of contact within the setting | Then this enacts access to and acceptance of the programme by patients and providers alike | Resulting in   - increased participation, - increased adherence - motivation to implement the intervention in a given setting | (5) (6) (9) |
| 4  Multidisciplinary team approach | If, in a multidisciplinary team,   - “everyone […] is valued for the contributions” ( (6), p. 132) and - responsibilities are clear | Then this enacts   - an understanding of roles, - mutual respect and support, - team-based decision-making, - common purpose | Resulting in   - better teamwork - more integrated care (avoiding “professionals working in silos” ( (6), p. 134)) - more efficient use of resources | (5) (6) |
| 5  Clear patient pathway | If there are   - specific entry points, - clear referral guidelines, and - a “clear allocation of responsibility in relation to […] referral and providing patients an optimised and transparent care pathway” ( (10), p. 16) | Then this enacts   - smooth referral of patients between disciplines - shared accountability | Resulting in   - maximisation of the benefits of prehabilitation - care integration - optimal use of resources | (1) (10) |

**Facilitators:**

**Well-timed and appropriate information provision**

Hirschhorn, Kolt (1):

“The timing of information, the method by which the information was given (e.g. verbally or in writing) and the relative emphasis placed upon the information, were all potential barriers or enablers to transfer of knowledge, and hence preoperative PFMT [pelvic floor muscle training].” (p. 7)

Francis-Coad, Edgar (2):

“Patients also expressed feelings of frustration and anxiety regarding hospital procedures, including repetitive gathering of information and poor communication across departments.” (p. 135)

“Patients also identified barriers in the volume, timing, type and way in which information was provided that affected their capability to optimise their recovery. The volume of new information was overwhelming and difficult to absorb for most patients in the pre-operative consults with health professional staff […]” (p.137)

“Patients who were placed on a waiting list from four weeks to four months found it difficult to remember the information they were given at the primary clinic appointments and felt that they required reminders prior to admission for their surgery […]” (p. 138)

“If designing a multimodal prehabilitation program, weekly sessions could be conducted and information delivered in manageable ‘chunks’. This requirement is supported by information processing theory that explains we are only able to process and commit to memory approximately five to seven pieces of information at one time point.” (p. 142)

Brown, Kenney (3):

“Education efforts should focus particularly on promoting patient engagement and maximizing weekly step count throughout the program.” (p. 27)

Boden, El-Ansary (4):

“An information booklet is not sufficient to provide information on early ambulation and breathing exercises […]” (p. 3)

“Experimental group participants expressed appreciation in the degree of detail provided by the physiotherapist with many finding it fascinating and intriguing. Because of this, they reported it easy to remember and differentiable from other information provided that day. Participants liked the use of pictures and this assisted in information recall.” (p. 11)

“Preoperative education leads to significantly better postoperative information recall and a small improvement in ambulation on the first postoperative day when compared to patients who received information via booklet alone. The preoperative physiotherapy education was highly memorable standing out from all other preadmission clinic information. Qualitative results attribute this to the personal delivery of detailed, interesting, and practical information that patients place high value upon.” (p. 13)

“In our trial the superior recall rate is most likely dependent on the type and mode of information provided rather than on the personality of the therapist who provided it.” (p. 14)

**Patient-centred individualisation**

Francis-Coad, Edgar (2):

“Ascertaining what is meaningful to patients in the preoperative, perioperative and postoperative periods may be challenging but is fundamental to executing patient-centred care in practice28 as engaging patients in their care has been associated with improved clinical outcomes and care experience.“ (p. 142)

“Patients identified a need for tailored exercise prescription” (p.141)

“Patients also felt that tailored nutrition plans pre-surgery would have been beneficial in effectively preparing surgery.” (p. 140)

Grimmett, Bradbury (5):

“Methods to increase self-efficacy to engage in the behaviours required of prehabilitation programs is likely to improve uptake and action.“ (p. 3)

“Ultimately, delivery mode decisions will depend on local context and should be based on a needs analysis and consultation with all relevant stakeholders, including the end users.” (p.2)

“[…] interventions developed with a person-centred approach, considering individual needs and preferences will increase patient engagement and are more effective than expert only design processes.” (p. 2)

Davis, Van Rooijen (6):

“Patients may have different degrees of risk for any given component of the intervention and therefore may require different levels of support for each element […]” (p. 131)

“’One prescription does not fit all’. There is a need to assess patients using validated metrics and plan appropriate individualized interventions to maximise the benefits and to control costs.” (p.132)

Beck, Vind Thaysen (7):

“[…] patients requested a still more tailored approach, based on individual needs.” (p. 148)

“[…] patients had the freedom to choose what suited them the best – rendering the preparation practicable and realistic.” (p.151)

“Even though findings from the interviews revealed that patients appreciated the freedom of the general recommendations, they still requested a more tailored approach provided by support from the health professionals.” (p. 151)

Boote, Newsome (7):

“Most patients found the bespoke physiotherapy intervention to be valuable. Those who responded positively appreciated the way it was tailored to their individual condition and some contrasted it favourably with physiotherapy that they had previously received, which was characterized as ‘tick box physiotherapy’ by one patient.” (p. 6)

**Integration into the setting**

Grimmett, Bradbury (5):

“Community based programs that offer more locally available support have also shown positive preliminary results.” (p. 2)

“Ultimately, delivery mode decisions will depend on local context and should be based on a needs analysis and consultation with all relevant stakeholders, including the end users.” (p.2)

Davis, Van Rooijen (6):

“Working together with a community centre could be useful when patients cannot or do not wish to travel.” (p. 133)

“Ensure the programme is feasible and fits your local situation […]” (p. 134)

Bates, West (9):

“Targeted interventions can take place safely within a community facility, but such a lifestyle change can present significant challenges to patients. Appointment-based, local and supervised facilities are likely to increase adherence.” (p. e12)

**Multidisciplinary team approach**

Grimmett, Bradbury (5):

“Furthermore, if an ambition of the program is to promote longer-term behavior change patients need to develop skills to engage in these behaviors autonomously. This may be at odds to a highly supervised and structured approach that may be favored by others in the team. By working together, an appropriate balance can be achieved and ultimately enhance program effectiveness.” (p. 3)

Davis, Van Rooijen (6):

“Prehabilitation requires a cohesive team where everyone in the team is valued for the contributions to the programme’s success. Each team member assesses the patient, and together, they recommend a prehabilitation prescription for each patient.” (p. 132)

“Collaboration with multiple stakeholders is key in prehabilitation. The right project lead is important to keep all believers linked together.” (p. 133)

“The patient journey needs to avoid professionals working in silos.” (p. 134)

**Clear patient pathway**

Hirschhorn, Kolt (1):

“Their ability to provide PFMT was still seen as contingent upon the patient having received a direct referral from the surgeon, this referral perceived as the key enabler.” (p. 6)

“Additional enablers to referral/provision of PFMT were referrers’/ providers’ perceptions of the psychological benefits (to patients) of having a ‘team approach’ to cancer care, of patients being adequately mentally prepared for PPUI, and knowing that they (the patients) had done all they could to prepare for radical prostatectomy.” (p. 9)

Husted, Bandholm (10):

“Clear allocation of responsibility in relation to exercise referral and providing patients an optimized and transparent care pathway becomes potential facilitators for the coordinated non-surgical and surgical care pathway.” (p.16)

“[…] physical therapists focus more on the care pathway as a whole. It becomes a facilitator for the physical therapists to contribute to the coordinated care pathway by providing exercise before the decision on surgery is taken.” (p. 19)

## Appendix E: Preliminary Programme Theories for Barriers to the Implementation of Prehabilitation into Routine Health Care

| **CMOC** | **Context** | **+ Mechanism** | **= Outcome** | **References** |
| --- | --- | --- | --- | --- |
| 1  Overwhelming and/or inadequate information provision | If information is provided   - at an inappropriate time, - in a non-engaging manner, - with the wrong emphasis /focus, and - if “the volume of new information was overwhelming and difficult to absorb” ( (2), p. 137) | Then this enacts   - overwhelming of patients, - no engagement and understanding for the benefit of the programme by the patient | Resulting in   - reluctance to participate, - no awareness of own role in improving pre-surgery, - difficulties in adherence, - no motivation | (1, 2, 4, 11, 12) |
| 2  Lack of multi-modality and/or adaptability | If the prehabilitation programme   - does not consist of adequate modalities, - does not include all necessary health care professionals, and - is not adaptable to the individual capabilities, needs and mobility of the patient, e.g., if it does not provide the “option of exercise adjustment or the addition of other exercises” ( (10), p. 9), and - is not adapted to the local setting | Then this enacts   - excessive demand on the patient (feeling overwhelmed), - causing extra stresses | Resulting in   - inability to participate or even access the intervention, - exclusion of patient groups in need | (5, 6, 13, 14) |
| 3  Fragmentation between providers | If providers   - do not value each member, - do not communicate well, - do not know their responsibilities, and - there are “mixed feelings and sometimes conflicting interest” ( (10), p. 19) between different disciplines along the care pathway | Then this   - disturbs the referral of patients, - enacts a lack of common purpose, - enacts no shared sense of accountability, - leads to loss of information | Resulting in   - difficulties in implementation, - Difficulties in maximising the benefits of the intervention, - lack of care integration | (1, 5, 10, 14) |
| 4  Resource constraints | If “availability and management of staffing, physical space and equipment” ( (1), p. 7) as well as funding and/or reimbursement of the prehabilitation programme are sparse | Then this enacts   - lack of motivation, - lack of acceptance for the implementation | Resulting in   - decreased job satisfaction, - exhaustion, - suboptimal and limited prehabilitation provision | (1, 14-16) |
| 5  Lack of (social) support | If patients are not supported by family, which have been reported to “[provide] the essential support that enabled patients to successfully complete the journey from pre-op to final recovery” ( (2), p. 139), or by friends, and the programme providers | Then this enacts   - emotional/psychological stress - lack of focus on the intervention | Resulting in   - distraction, - difficulties in compliance/adherence, - limited success, and - non-participation/drop-out of patients | (2, 7, 17, 18) |

**Barriers:**

**Overwhelming Information Provision**

Hirschhorn, Kolt (1):

“The timing of information, the method by which the information was given (e.g. verbally or in writing) and the relative emphasis placed upon the information, were all potential barriers or enablers to transfer of knowledge, and hence preoperative PFMT.” (p. 7)

“Patients also varied considerably in their preferences for information (volume and mode of delivery) about prostate cancer and related issues, including PFMT. Some preferred written information that could be reviewed at home, others felt that ‘too much information’ was presented in this way, and thus was likely to be ignored.” (p. 9)

Francis-Coad, Edgar (2):

“Some pre-op appointments provided too much information at one time leaving patients overwhelmed: Patients identified they need information provided in ‘manageable chunks’ for assimilation” (p. 141)

Boden, El-Ansary (4):

“Many patients admitted to not reading the booklet with some saying they received so much written literature it was difficult to differentiate one booklet from another. Additionally, a number of participants reported difficulty reading and preferred personal delivery of information with pictorial content.” (p. 11)

“A number of control group participants felt overwhelmed during a long preadmission clinic and attributed this to not being able to remember information.” (p. 12)

McLaughlin, Palmer (11):

“Communicating the nature and purpose of the programme to patients was challenging and consequently the quality of the explanation received and understanding gained by patients was generally low.” (p.2)

Zhu, Moffa (12):

“Throughout prehabilitation, clinicians present patients with diagnosis, prognosis, and treatment option information which can be confusing for some patients.” (p. 4)

“Patients note various factors which cause them to feel dissatisfied with clinical care, including but not limited to: […] lack of clear and comprehensive information during the healthcare process […]” (p. 6)

**Lack of Adaptability**

Grimmett, Bradbury (5):

“Changing health behaviours is complex and requires much more than provision of information. Interventions that seek to support individual behaviour change are most effective when

they draw on behavioural science (National Institute of Clinical Excellence (NICE), 2014). Furthermore, interventions developed with a person-centred approach, considering individual needs and preferences will increase patient engagement and are more effective than expert only design processes (Trischler et al., 2018).” (p. 2)

Davis, Van Rooijen (6):

“The Person Based Approach (PBA) is particularly relevant to prehabilitation. The key focus of which is to gain ‘insight into how different people in different situations perceive and execute the behavioural elements of the intervention, why some elements may be particularly necessary or salient to them—or alternatively may be aversive or problematic—and thus how the intervention can be made more attractive, persuasive and feasible to implement’.“ (p. 134)

Ismail, Cormie (13):

“[…] scalability for effective prehabilitation programs is a major challenge […]” (p. 378)

Shukla, Granger (14):

“Finally, it is important to explore alternate means of delivering prehabilitation for patients with lung cancer, such as via telehealth with virtual/online exercise platforms, which could allow delivery of programs to patients in their home setting. A home-based model would be appealing, particularly for patients who are unable to access the traditional center-based programs who often report barriers such as living too far away from the center or lack of transport.” (p. 5)

**Fragmentation Between Providers**

Hirschhorn, Kolt (1):

“[…] no such system of referral existed in the public urology clinic.” (p. 8)

“The pathway today, we find that it's a little bit difficult is, once the patient get's onto (a) specialist's care, we find often we don't see the patient coming back to us for a little while, and quite often it's… already (having had) a prostatectomy.” (p. 4)

“I just don't get the referrals to see (men for PFMT), which is probably because doctors are not aware that I provide the treatment, which is again, probably because I never publicise that I provide it.” (p. 4)

“[…] surgeons claimed to be unaware of their colleagues’ referral practices.” (p. 9)

Grimmett, Bradbury (5):

“As with all multidisciplinary teams, there can be tensions between disciplines. For example, the clinical team may be focused on the optimal intervention or stimulus for increasing cardiorespiratory fitness prior to surgery, whereas the behavioral expert may prioritize the optimal intervention to maximize motivation.” (p. 3)

Husted, Bandholm (10):

“Referring to exercise without knowing the content of the treatment provide and the time associated with referral to exercise becomes potential barriers for the orthopedic surgeon to refer patients to exercise.” (p. 15)

“[…] orthopedic surgeons express frustration with variation in the treatment provided for the patients when they refer them to exercise in the municipality which becomes a barrier to referring patients to exercise. The physical therapists are positive in respect of the coordinated care pathway as they believe this will mean that patients are provided with quality care.” (p. 16)

“The experience of loss of control challenges their profession role and becomes a barrier for implementing home-based exercise therapy with one exercise.” (p. 17)

Shukla, Granger (14):

“The respondents reported that prehabilitation programs were either not accessible, or available and difficult to refer to. The main barriers to prehabilitation recorded were access to allied health professionals (57%) […]” (p.3)

“The challenges for prehabilitation that must be overcome include […] establishing strong referral pathways, developing good relationships with the surgeon gatekeepers […]” (p. 4).

**Resource Constraints**

Hirschhorn, Kolt (1):

“Perceptions of financial cost of private sector PFMT, limited knowledge amongst referrers of public sector providers of PFMT, and limited awareness amongst patients of the benefits of PFMT were all posited to contribute to suboptimal PFMT provision and receipt.” (p. 1)

Shukla, Granger (14):

“Barriers to the implementation of robust prehabilitation programs for patients with lung cancer include the traditional hurdles of funding […]” (p. 2)

“The main barriers to prehabilitation recorded were […] costs/financial constraints (33%).” (p.3)

Drummond, Lambert (15):

“[…] challenges remain in its seamless implementation. Notably, hurdles to overcome include […] the initial costs of acquiring the new technologies.” (p. 7)

Norris and Close (16):

“[…] only a minority of institutions have implemented prehabilitation programs despite the majority of surgeons being prepared to delay surgery in appropriate patients if deemed of benefit. In part, this is likely to be due to the capacity of hospitals to provide resources to deliver this intervention but also due to a lack of assessment of functional status and access to services that may allow them to perform this formally, such as cardiopulmonary exercise testing.” (p. 1525)

**Lack of (Social) Support**

Francis-Coad, Edgar (2):

“Family members were not always present at hospital appointments when information was provided to the patient and were thus left to their own devices, this sometimes resulted in them seeking information from potentially unreliable sources.” (p. 138)

Beck, Vind Thaysen (7):

“[…] not all patients had a supportive social network, and strains within the family might impact self-care negatively, leading to vulnerability and a need of extra support from health professionals.” (p. 151)

“[…] family gatherings, work, practical activities and illness in the family could also be perceived as impediments to preparation.” (p. 152)

Catho, Guigard (17):

“Living alone, polypharmacy and a long delay before starting the rehabilitation programme were the main factors associated with the risk of not completing the programme.” (p. 5)

“Several studies have shown that family support and encouragement enhance participation and adherence to pulmonary rehabilitation particularly when the partner is able to participate in the patient’s care. In the present study, all but one of the participants who completed the full programme lived with a partner […]” (p. 6)

Moore, Scoggins (18):

“Many patients lack the understanding or environmental support to participate in a pre-habilitation program three to four times a week.” (p.2076)

## Appendix F: Excluded Articles After Full-Text Screening

| **Authors** | **Title** | **Year** | **Language** | **Exclusion criterium** |
| --- | --- | --- | --- | --- |
| Abdelmasih, Monica and Forbes, Thomas L. and Kayssi, Ahmed (19) | Prehabilitation Prior to Surgery: A Scoping Review and Pilot Quality Improvement Project | 2021 | English | P: not focused on frailty |
| Anonymous (20) | Response to "Prehabilitation for Patients With Esophageal Cancer During Neoadjuvant Treatment and Surgery" | 2019 | English | P: not focused on frailty |
| Chen, H. and Ferrando, A. A. and White, M. G. and Pauly, M. and Bartter, T. and Dunn, M. A. and Kim, W. R. and Duarte-Rojo, A. (21) | A randomized pilot trial of home-based physical activity plus dietary intervention to improve physical function in patients with advanced liver disease | 2019 | English | P: not focused on frailty |
| Fromm, C. G. & Metzler, D. J. (22) | Preparing your older patient for surgery | 1993 | English | P: not focused on frailty |
| Gillis, C. and Ljungqvist, O. and Carli, F. (23) | Prehabilitation, enhanced recovery after surgery, or both? A narrative review | 2022 | English | P: not focused on frailty |
| Halliday, L. J. and Doganay, E. and Wynter-Blyth, V. A. and Hanna, G. B. and Moorthy, K. (24) | The Impact of Prehabilitation on Post-operative Outcomes in Oesophageal Cancer Surgery: a Propensity Score Matched Comparison | 2021 | English | P: not focused on frailty |
| Horattas, I. and Visioni, A. and Horattas, M. C. (25) | Improving Surgical Outcomes Prior to Incision: Superior Outcomes in the Geriatric Surgery Population after Prehabilitation | 2020 | English | P: not focused on frailty |
| Kaye, D. R. and Thelen-Perry, S. and Schafer, C. and Qin, Y. and Reger, H. I. and Parker, C. and Kaffenberg, S. and Herrel, L. and Morgan, T. and Weizer, A. and Hafez, K. and Montgomery, J. S. (26) | The feasibility, safety and impact of a prehabilitation program for patients undergoing cystectomy | 2019 | English | P: not focused on frailty |
| Palmer, J. and Pymer, S. and Smith, G. E. and Harwood, A. E. and Ingle, L. and Huang, C. and Chetter, I. C. (27) | Presurgery exercise-based conditioning interventions (prehabilitation) in adults undergoing lower limb surgery for peripheral arterial disease (Cochrane review) [with consumer summary] | 2020 | English | P: not focused on frailty |
| Pang, N. Q. and Tan, Y. X. and Samuel, M. and Tan, K. K. and Bonney, G. K. and Yi, H. and Kow, W. C. A. (28) | Multimodal prehabilitation in older adults before major abdominal surgery: a systematic review and meta-analysis | 2022 | English | P: not focused on frailty |
| Rapti, A. and Kerenidi, T. and Zarogoulidis, K. (29) | Treatment of lung cancer | 2012 | English | P: not focused on frailty |
| Santa Mina, D. and Alibhai, S. M. H. (30) | Prehabilitation in geriatric oncology | 2020 | English | P: not focused on frailty |
| Slim, K. and Selvy, M. and Theissen, A. (31) | Prehabilitation before major surgery: growing interest but persistent haze | 2021 | English | P: not focused on frailty |
| Wynne, S. and Dickinson, F. and Fraser, S. and Peat, N. and Labuc, P. and Bracegirdle, R. and Hawley, E. (32) | OA08.04 Providing Thoracic Prehabilitation during COVID-19: Review of a Virtual Model | 2021 | English | P: not focused on frailty |
| Argillander, T. E. and van der Zanden, V. and van der Zaag-Loonen, H. J. and Paarlberg, K. M. and Meijer, W. J. and Kruse, A. J. and van Westreenen, H. L. and van Duijvendijk, P. and Mourits, M. J. E. and van Munster, B. C. (33) | Preoperative physical activity and frailty in older patients undergoing cancer surgery - PREsurgery study | 2022 | English | I: definition of prehabilitation not fulfilled |
| Buttery, A. K. (34) | Cardiac Rehabilitation for Frail Older People | 2020 | English | I: definition of prehabilitation not fulfilled |
| Czobor, N. R. and Lehot, J. J. and Holndonner-Kirst, E. and Tully, P. J. and Gal, J. and Szekely, A. (35) | Frailty in patients undergoing vascular surgery: A narrative review of current evidence | 2019 | English | I: definition of prehabilitation not fulfilled |
| Daniel, K. M. and Ray, C. and Cason, C. (36) | Progressive functional wii-hab in pre-frail older adults | 2011 | English | I: definition of prehabilitation not fulfilled |
| Daniels, S. and Lee, M. and Moug, S. and Wilson, T. and Burton, M. and Brown, S. and Wyld, L. (37) | Semi-structured healthcare professional interviews to explore their preferences for the assessment and optimisation of older adults facing major gastrointestinal surgery | 2021 | English | I: definition of prehabilitation not fulfilled |
| Ettema, R. and Schuurmans, M. J. and Schutijser, B. and van Baar, M. and Kamphof, N. and Kalkman, C. J. (38) | Feasibility of a nursing intervention to prepare frail older patients for cardiac surgery: a mixed-methods study | 2015 | English | I: definition of prehabilitation not fulfilled |
| Forcillo, J. and Perrault, L. P. (39) | If too frail, functional benefit following cardiac surgery may fail: A role for prehabilitation? | 2017 | English | I: definition of prehabilitation not fulfilled |
| Gill, T. M. and Baker, D. I. and Gottschalk, M. and Peduzzi, P. N. and Allore, H. and Van Ness, P. H. (40) | A prehabilitation program for the prevention of functional decline: Effect on higher-level physical function | 2004 | English | I: definition of prehabilitation not fulfilled |
| Goh, S. S. N. and Chia, C. L. K. (41) | Improving outcomes in geriatric surgery: Is there more to the equation? | 2022 | English | I: definition of prehabilitation not fulfilled |
| Griebling, T. L. (42) | Re: Accumulated frailty characteristics predict postoperative discharge institutionalization in the geriatric patient | 2011 | English | I: definition of prehabilitation not fulfilled |
| Lin, F. P. and Bloomer, P. M. and Grubbs, R. and Rockette-Wagner, B. and Tevar, A. D. and Forman, D. E. and Dunn, M. A. and Duarte-Rojo, A. (43) | Low daily step count from a personal activity tracker is associated with a higher risk for hospital admission and death in community-dwelling patients with cirrhosis | 2021 | English | I: definition of prehabilitation not fulfilled |
| Pellathy, T. P. and Rodriguez, K. L. and Hruska, K. L. and Kennedy, K. A. and Hall, D. E. (44) | Shared Decision Making in High Risk Settings: Frail Patients' Perceptions of Preoperative Goal Clarification Consultation By Palliative Care Specialists | 2021 | English | I: definition of prehabilitation not fulfilled |
| Puts, M. T. E. and Toubasi, S. and Andrew, M. K. and Ashe, M. C. and Ploeg, J. and Atkinson, E. and Ayala, A. P. and Roy, A. and Monforte, M. R. and Bergman, H. and McGilton, K. (45) | Interventions to prevent or reduce the level of frailty in community-dwelling older adults: A scoping review of the literature and international policies | 2017 | English | I: definition of prehabilitation not fulfilled |
| Baimas-George, M. and Watson, M. and Thompson, K. and Shastry, V. and Iannitti, D. and Martinie, J. B. and Baker, E. and Parala-Metz, A. and Vrochides, D. (46) | Prehabilitation for Hepatopancreatobiliary Surgical Patients: Interim Analysis Demonstrates a Protective Effect From Neoadjuvant Chemotherapy and Improvement in the Frailty Phenotype | 2021 | English | O a): no information on facilitators/barriers |
| Bojesen, R. D. and Jørgensen, L. B. and Grube, C. and Skou, S. T. and Johansen, C. and Dalton, S. O. and Gögenur, I. (47) | Fit for Surgery-feasibility of short-course multimodal individualized prehabilitation in high-risk frail colon cancer patients prior to surgery | 2022 | English | O a): no information on facilitators/barriers |
| Calabro, M. and Bonasso, C. and Pipitone Federico, N. S. and Cuzzola, B. and Spidalieri, L. and Danna, R. and Muratore, A. (48) | A preoperative training program | 2020 | English | O a): no information on facilitators/barriers |
| Carli, F. and Brown, R. and Kennepohl, S. (49) | Prehabilitation to enhance postoperative recovery for an octogenarian following robotic-assisted hysterectomy with endometrial cancer | 2012 | English | O a): no information on facilitators/barriers |
| Carli, F. and Awasthi, R. and Gillis, C. and Kassouf, W. (50) | Optimizing a frail elderly patient for radical cystectomy with a prehabilitation program | 2014 | English | O a): no information on facilitators/barriers |
| Carli, F. and Bousquet-Dion, G. and Awasthi, R. and Elsherbini, N. and Liberman, S. and Boutros, M. and Stein, B. and Charlebois, P. and Ghitulescu, G. and Morin, N. and Jagoe, T. and Scheede-Bergdahl, C. and Minnella, E. M. and Fiore, J. F., Jr. (51) | Effect of multimodal prehabilitation versus postoperative rehabilitation on 30-day postoperative complications for frail patients undergoing resection of colorectal cancer: a randomized clinical trial [with consumer summary] | 2020 | English | O a): no information on facilitators/barriers |
| Carli, F. and Bousquet-Dion, G. and Fiore, J. F. (52) | Prehabilitation vs Postoperative Rehabilitation for Frail Patients-Reply | 2020 | English | O a): no information on facilitators/barriers |
| Chia, C. L. K. and Mantoo, S. K. and Tan, K. Y. (53) | "Start to finish transinstitutional transdisciplinary care": A novel approach improves colorectal surgical outcomes in elderly patients | 2015 | English | O a): no information on facilitators/barriers |
| Cooper, L. and Frain, L. and Jaklitsch, M. T. (54) | Prehabilitation vs Postoperative Rehabilitation for Frail Patients | 2020 | English | O a): no information on facilitators/barriers |
| Derck, J. and Klemptner, E. and Mazurek, A. and Fakhoury, J. and Sonnenday, C. (55) | Prehabilitation for patients with end-stage liver disease | 2015 | English | O a): no information on facilitators/barriers |
| Dholakia, J. and Cohn, D. E. and Straughn, J. M. and Dilley, S. E. (56) | Prehabilitation for medically frail patients undergoing surgery for epithelial ovarian cancer: a cost-effectiveness analysis | 2021 | English | O a): no information on facilitators/barriers |
| Dworsky, J. Q. and Castle, S. C. and Lee, C. C. and Singh, S. P. and Russell, M. M. (57) | Gerofit Prehabilitation Pilot Program: Preparing Frail Older Veterans for Surgery | 2019 | English | O a): no information on facilitators/barriers |
| Ellenberger, C. and Schorer, R. and Bedat, B. and Hagerman, A. and Triponez, F. and Karenovics, W. and Licker, M. (58) | How can we minimize the risks by optimizing patient's condition shortly before thoracic surgery? | 2021 | English | O a): no information on facilitators/barriers |
| Gillis, C. and Carli, F. (59) | Patients with poor functional walking capacity experience significantly more medical complications post-colorectal surgery than those with higher functional walking capacity | 2021 | English | O a): no information on facilitators/barriers |
| Gillis, C. and Fenton, T. R. and Gramlich, L. and Sajobi, T. T. and Culos-Reed, S. N. and Bousquet-Dion, G. and Elsherbini, N. and Fiore, J. F. and Minnella, E. M. and Awasthi, R. and et al. (60) | Older frail prehabilitated patients who cannot attain a 400 m 6-min walking distance before colorectal surgery suffer more postoperative complications | 2021 | English | O a): no information on facilitators/barriers |
| Grimes, L. and Outtrim, J. G. and Griffin, S. J. and Ercole, A. (61) | Accelerometery as a measure of modifiable physical activity in high-risk elderly preoperative patients: a prospective observational pilot study | 2019 | English | O a): no information on facilitators/barriers |
| Gritsenko, K. and Helander, E. and Webb, M. P. K. and Okeagu, C. N. and Hyatali, F. and Renschler, J. S. and Anzalone, F. and Cornett, E. M. and Urman, R. D. and Kaye, A. D. (62) | Preoperative frailty assessment combined with prehabilitation and nutrition strategies: Emerging concepts and clinical outcomes | 2020 | English | O a): no information on facilitators/barriers |
| Hanna, K. and Ditillo, M. and Joseph, B. (63) | The role of frailty and prehabilitation in surgery | 2019 | English | O a): no information on facilitators/barriers |
| Ji, Y. D. and Dodson, T. B. (64) | Minimizing Frailty Preoperatively Through Prehabilitation: Improving Surgical Outcomes | 2022 | English | O a): no information on facilitators/barriers |
| Kako, J. and Kajiwara, K. and Tatematsu, N. (65) | Prehabilitation vs Postoperative Rehabilitation for Frail Patients | 2020 | English | O a): no information on facilitators/barriers |
| Kaplan, J. A. and Brinson, Z. and Hofer, R. and O'Sullivan, P. and Chang, A. and Horvath, H. and Chang, G. J. and Finlayson, E. (66) | Early learners as health coaches for older adults preparing for surgery | 2017 | English | O a): no information on facilitators/barriers |
| Keller, D. S. and Carter, B. and Moug, S. J. (67) | Prehabilitation vs Postoperative Rehabilitation for Frail Patients | 2020 | English | O a): no information on facilitators/barriers |
| Loh, H. and Foo, F. and Tan, W. and Sivarajah, S. and Chew, M. (68) | Programme for enhanced elderly recovery at sengkang (PEERS) | 2020 | English | O a): no information on facilitators/barriers |
| Lorenz, E. and Hickson, L. and Weatherly, R. and Thompson, K. and Hogan, M. and Kennedy, C. (69) | The impact of 4-weeks of supervised exercise on frailty and lower extremity (LE) function in patients with advanced chronic kidney disease (CKD) | 2021 | English | O a): no information on facilitators/barriers |
| Magnano, M. and Andreis, M. and Nazionale, G. and Mola, P. and Machetta, G. (70) | The role of prehabilitation in frail patient with a head and neck cancer | 2021 | English | O a): no information on facilitators/barriers |
| Milder, D. A. and Pillinger, N. L. and Kam, P. C. A. (71) | The role of prehabilitation in frail surgical patients: a systematic review | 2018 | English | O a): no information on facilitators/barriers |
| Mrdutt, M. M. and Papaconstantinou, H. T. and Robinson, B. D. and Bird, E. T. and Isbell, C. L. (72) | Re: Preoperative frailty and surgical outcomes across diverse surgical subspecialties in a large health care system | 2019 | English | O a): no information on facilitators/barriers |
| Ngo-Huang, A. and Fontillas, R. C. and Gupta, E. and Sahai, S. K. and Popovich, S. and Andrabi, T. and French, K. E. (73) | Implementing prehabilitation as part of enhanced recovery after surgery (ERAS) efforts at a comprehensive cancer center: A team-based approach | 2018 | English | O a): no information on facilitators/barriers |
| Rafique, H. and Worley, G. and Anele, C. and Askari, A. and Faiz, O. (74) | The use of prehabilitation in those undergoing colorectal surgery: A systematic review | 2017 | English | O a): no information on facilitators/barriers |
| Ramírez-Martín, R. and Pérez-Rodríguez, P. and Menéndez-Colino, R. and Martín Maestre, I. and Gazo Martínez, J. A. and Marijuán Martín, J. L. and Alarcón Alarcón, T. and Díez Sebastián, J. and González-Montalvo, J. I. (75) | Prehabilitation and perioperative geriatric care in patients aged over 80 years with colorectal cancer: Results of a cross-speciality geriatrics program | 2022 | English | O a): no information on facilitators/barriers |
| Resnick, M. J. (76) | Re: effect of Multimodal Prehabilitation vs Postoperative Rehabilitation on 30-Day Postoperative Complications for Frail Patients Undergoing Resection of Colorectal Cancer: a Randomized Clinical Trial | 2020 | English | O a): no information on facilitators/barriers |
| Rostoft, S. (77) | Improving the care for older patients with cancer | 2015 | English | O a): no information on facilitators/barriers |
| Salvi, R. and Meoli, I. and Cennamo, A. and Perrotta, F. and Saverio Cerqua, F. and Montesano, R. and Curcio, C. and Lassandro, F. and Stefanelli, F. and Grella, E. and Tafuri, D. and Mazzarella, G. and Bianco, A. (78) | Preoperative high-intensity training in frail old patients undergoing pulmonary resection for NSCLC | 2016 | English | O a): no information on facilitators/barriers |
| Siu, A. T. Y. and Poulton, T. and Ismail, H. and Riedel, B. and Dhesi, J. (79) | Prehabilitation in the Older People: Current Developments | 2021 | English | O a): no information on facilitators/barriers |
| Söhle, M. and Coburn, M. (80) | [Perioperative Medicine in Visceral Surgery in the Elderly Patient from an Anaesthesiological Perspective] | 2021 | German | O a): no information on facilitators/barriers |
| Van Der Hulst, H. and Bastiaannet, E. and van der Bol, J. and Dekker, J. W. (81) | Can prehabilitation protect frail colorectal cancer patients over 70 years old from complications after surgery? | 2021 | English | O a): no information on facilitators/barriers |
| van der Hulst, H. C. and Bastiaannet, E. and Portielje, J. E. A. and van der Bol, J. M. and Dekker, J. W. T. (82) | Can physical prehabilitation prevent complications after colorectal cancer surgery in frail older patients? | 2021 | English | O a): no information on facilitators/barriers |
| van der Vlies, E. and Smits, A. B. and Los, M. and van Hengel, M. and Bos, W. J. W. and Dijksman, L. M. and van Dongen, E. P. A. and Noordzij, P. G. (83) | Implementation of a preoperative multidisciplinary team approach for frail colorectal cancer patients: Influence on patient selection, prehabilitation and outcome | 2020 | English | O a): no information on facilitators/barriers |
| Van Wijk, L. and Buis, C. I. and Klaase, J. M. (84) | Feasibility of a prehabilitation clinic for patients undergoing oncologic abdominal surgery: the FRAIL study | 2020 | English | O a): no information on facilitators/barriers |
| Visina, J. M. and Bloomer, P. M. and Lin, F. P. and Dunn, M. A. and Josbeno, D. A. and Tevar, A. D. and Hughes, C. and Jakicic, J. M. and Duarte-Rojo, A. (85) | Prehabilitation adherence as predictor for improved physical function in cirrhosis | 2020 | English | O a): no information on facilitators/barriers |
| Waite, I. and Deshpande, R. and Baghai, M. and Massey, T. and Wendler, O. and Greenwood, S. (86) | Home-based preoperative rehabilitation (prehab) to improve physical function and reduce hospital length of stay for frail patients undergoing coronary artery bypass graft and valve surgery | 2017 | English | O a): no information on facilitators/barriers |
| Yau, K. W. and Underwood, M. J. and Joynt, G. M. and Lee, A. (87) | Determinants of exercise intensity achieved during exercise prehabilitation program for patients awaiting cardiac surgery | 2020 | English | O a): no information on facilitators/barriers |
| Yau, K. W. D. and Underwood, M. and Joynt, G. and Lee, A. (88) | Exercise prehabilitation (Prequel) for patients undergoing cardiac surgery: Preliminary results | 2021 | English | O a): no information on facilitators/barriers |
| Cheng, X. S. and Myers, J. N. and Chertow, G. M. and Rabkin, R. and Chan, K. N. and Chen, Y. and Tan, J. C. (89) | Prehabilitation for kidney transplant candidates: Is it time? | 2017 | English | O b): mere call for future research |
| Lorenz, E. C. and Hickson, L. J. and Weatherly, R. M. and Thompson, K. L. and Walker, H. A. and Rasmussen, J. M. and Stewart, T. L. and Garrett, J. K. and Amer, H. and Kennedy, C. C. (90) | Protocolized exercise improves frailty parameters and lower extremity impairment: A promising prehabilitation strategy for kidney transplant candidates | 2020 | English | O b): mere call for future research |
| Michalik, C. and Maciukiewicz, P. and Drewa, T. and Kenig, J. and Juszczak, K. (91) | Frailty, geriatric assessment and prehabilitation in elderly patients undergoing urological surgery - is there a need for change of the daily clinical practice? Synthesis of the available literature | 2020 | English | O b): mere call for future research |
| Oosting, E. and Hoogeboom, T. and Appelman, S. and Dronkers, J. and Van Meeteren, N. (92) | Feasibility of an intensive therapeutic exercise program for frail elderly prior to total hip arthroplasty: two randomized pilot studies | 2015 | English | O b): mere call for future research |
| Baimas-George, M. and Watson, M. and Elhage, S. and Parala-Metz, A. and Vrochides, D. and Davis, B. R. (93) | Prehabilitation in frail surgical patients: a systematic review | 2020 | English | O c): only stating the existence of facilitators/barriers |
| McIsaac, D. I. and Hladkowicz, E. and Bryson, G. L. and Forster, A. J. and Gagne, S. and Huang, A. and Lalu, M. and Lavallee, L. T. and Moloo, H. and Nantel, J. and Power, B. and Scheede-Bergdahl, C. and van Walraven, C. and McCartney, C. J. L. and Taljaard, M. (94) | Home-based prehabilitation with exercise to improve postoperative recovery for older adults with frailty having cancer surgery: the PREHAB randomised clinical trial | 2022 | English | O c): only stating the existence of facilitators/barriers |
| Norris, C. M. and Close, J. C. T. (16) | Prehabilitation for the Frailty Syndrome: Improving Outcomes for Our Most Vulnerable Patients | 2020 | English | O c): only stating the existence of facilitators/barriers |
| van Munster, B. (95) | PREHABILITATION IN PREOPERATIVE CARE OF PREHAB AS MEDICINE | 2019 | English | O c): only stating the existence of facilitators/barriers |
| van Wijk, L. and van der Schnee, L. and Buis, C. I. and Hentzen, J. E. K. R. and Haveman, M. E. and Klaase, J. M. (96) | Reengineering the Preoperative Care Path to Implement a Prehabilitation Outpatient Clinic for Patients Referred for Hepatobiliary and Pancreatic Surgery | 2021 | English | O c): only stating the existence of facilitators/barriers |
| Shovel, L. and Dunne, J. and Whibley, J. and Fernandes, A. and Kasivisvanathan, R. (97) | A tertiary cancer centre experience of prehabilitation for surgical ovarian cancer patients receiving neoadjuvant chemotherapy: The royal Mile - Marsden integrated lifestyle and exercise programm | 2019 | English | O c): only stating the existence of facilitators/barriers |
| Actrn (98) | A randomised clinical trial comparing preoperative exercise in the home, hospital, and community with standard care in adults awaiting for major abdominal surgery | 2022 | English | S: ongoing study |
| Janssen, T. L. and Mosk, C. A. and van Hoof-de Lepper, C. C. H. A. and Wielders, D. and Seerden, T. C. J. and Steyerberg, E. W. and van Gammeren, A. J. and de Lange, D. C. and van Alphen, R. and van der Zee, M. and de Bruijn, R. M. and de Vries, J. and Wijsman, J. H. and Ho, G. H. and Gobardhan, P. D. and van der Laan, L. (99) | A multicomponent prehabilitation pathway to reduce the incidence of delirium in elderly patients in need of major abdominal surgery: study protocol for a before-and-after study | 2019 | English | S: ongoing study |
| Lindback, Y. and Tropp, H. and Enthoven, P. and Abbott, A. and Oberg, B. (100) | PREPARE: pre-surgery physiotherapy for patients with degenerative lumbar spine disorder: a randomized controlled trial | 2017 | English | S: ongoing study |
| Negm, A. M. and Kennedy, C. C. and Ioannidis, G. and Gajic-Veljanoski, O. and Lee, J. and Thabane, L. and Adachi, J. D. and Marr, S. and Lau, A. and Atkinson, S. et al. (101) | Getting fit for hip and knee replacement: a protocol for the Fit-Joints pilot randomized controlled trial of a multi-modal intervention in frail patients with osteoarthritis | 2018 | English | S: ongoing study |
| Ntr (102) | Prehabilitation for bowel cancer patients undergoing surgery to improve fitness and reduce complications | 2016 | English | S: ongoing study |
| Santa Mina, D. and Sellers, D. and Au, D. and Alibhai, S. M. H. and Clarke, H. and Cuthbertson, B. H. and Darling, G. and El Danab, A. and Govindarajan, A. and Ladha, K. and Matthew, A. G. and McCluskey, S. and Ng, K. A. and Quereshy, F. and Karkouti, K. and Randall, I. M. (103) | A Pragmatic Non-Randomized Trial of Prehabilitation Prior to Cancer Surgery: Study Protocol and COVID-19-Related Adaptations | 2021 | English | S: ongoing study |
| Brown, R. and Carli, F. and Kennepohl, S. (104) | Prehabilitation for a frail elderly patient with endometrial cancer: A case report | 2012 | English | duplicate missed in earlier screening |
| Carli, F. and Bousquet-Dion, G. and Awasthi, R. and Elsherbini, N. and Liberman, S. and Boutros, M. and Stein, B. and Charlebois, P. and Ghitulescu, G. and Morin, N. and Jagoe, T. and Scheede-Bergdahl, C. and Minnella, E. M. and Fiore, J. F., Jr. (105) | Effect of Multimodal Prehabilitation vs Postoperative Rehabilitation on 30-Day Postoperative Complications for Frail Patients Undergoing Resection of Colorectal Cancer: A Randomized Clinical Trial | 2020 | English | duplicate missed in earlier screening |
| Debes, C. and Aissou, M. and Beaussier, M. (106) | [Prehabilitation. Preparing patients for surgery to improve functional recovery and reduce postoperative morbidity] | 2014 | French | foreign language |
| Liu, C. and Zhu, M. (107) | Progress of researches on application of multimodal prehabilitation technique in geriatric surgery | 2020 | Chinese | foreign language |
| Duerksen, J. R. (108) | Hip fractures: special considerations for the elderly patient | 1982 | English | full-text not accessible |
| Eckmann, L. (109) | Surgery in the old age (author's transl) | 1977 | German | full-text not accessible |
| Rosenberg, J. (110) | Plan ahead to improve surgical outcomes for the elderly | 1998 | English | full-text not accessible |

P = Population = focus not on frail patients; I = Intervention = definition of prehabilitation not fulfilled; C = Comparison/Control, not applicable; O = outcome, here: information on facilitators/barriers – a) no information on facilitators/barriers, b) mere call for future research, c) only stating the existence of facilitators/barriers; S = study design, here: study design criteria not fulfilled, e.g., ongoing study

## Appendix G: Quotations supporting the refined programme theories

**Facilitators:**

**Well-timed and appropriate information provision**

Singer, Soong (114):

“The participant experience in the intervention was nearly uniformly positive. Even older participants found the app interface intuitive and user-friendly.” (p. 7)

Balagué and Arroyo (115):

“patients who have followed the preparation guidelines are more ‘obedient’ when it comes to complying with postoperative rehabilitation instructions, and they are more active in their recovery. One possible reason is that many of them had internalized that there are tasks that they were no longer to perform and, during the pre-rehabilitation process, they observed that they were able to partially or totally recover some of these capabilities. They become aware that progress is up to them, and they come to ‘enjoy’ the process. They also do not want to lose any progress made and, consequently want to recover it after surgery. Thus, this process not only prepares patients to better overcome surgery, but they also learn to acquire healthy lifestyle habits that are maintained over time.” (p. 176)

Agasi-Idenburg, Zuilen (117):

“[…] understandable information provided by a physician […] facilitate exercise.” (p. 1)

“To engage frail older adults with CRC in preoperative exercise program information on exercise should improve. (p. 1)

“The themes that emerged as facilitators of exercise were understanding its benefits and having a history of exercising in the past.” (p. 4)

“If the goal is to stimulate exercise of CRC patients as part of preparation for surgery, priority should be given to facilitating physicians’ involvement in informing patients about the value of physical activity and the need to exercise.” (p. 5)

Agasi-Idenburg, Koning-van Zuilen (116):

“It is important that patients understand the potential benefits for PA-Prehab [*physical-activity based*] (EP) [*elderly patients*], and are rewarded for their efforts (IC) [*informal* *caregivers*].”(p. S61)

Lin, Visina (122):

“Transplant providers and patients need to work together under a common understanding that attendance to prehabilitation sessions and adherence to exercise prescription are key to the candidate’s well-being and survival during the waitlist period and early after LT [*liver* *transplantation*].” (p. 2114)

Carli, Bessissow (135):

“The importance of education and empowerment cannot be understated.” (p. 322)

“It is helpful when the surgeon explains the concept before a patient is seen in consultation, since it helps demystify the reason for the intervention. As with any other medical test or procedure, patients are more likely to consent to being seen for preoperative treatment when they are anticipating the consultation and understand its benefits. It is especially helpful to refer the patient at the first visit so that the timeline to surgical or other care can be respected without undue delays.” (p. 324).

Afilalo (142):

“One of the most impactful strategies to increase participation in CR [*cardiac* *rehabilitation*] is for the treating physician to explain and strongly recommend CR to the patient and family.” (p. 449)

“ […] patients were most responsive and incentivized when the benefits of CR were framed as ‘helping to perform ADLs *[activities of daily living*] as long as possible’ and ‘contributing to healthy aging’ (rather than decreasing reinfarction or mortality rates).” (p. 449)

Heil, Driessen (143):

“[…] patients are often unaware of the impact of surgery on their physical and mental condition and therefore, creating awareness of this impact is a facilitating factor.” (p. 11)

“Previous studies, interviewing patients, highlighted already the importance of appropriate information provision […]” (p. 12)

McAdams-DeMarco, Ying (120):

“[…] participants felt that prehabilitation helped them become more physical and mentally prepared for transplantation.” (p. 5)

**Patient-centred individualisation**

Feng, Dorrance (111):

“[…] program was easy to follow and well-suited to be completed at home” (p. 180)

Agasi-Idenburg, Zuilen (117)

“Exercise programs should be easily accessible and take personal preferences, needs and abilities into account.” (p. 1)

“Exercise should not be too intensive (Patients, ICs) and should be adjusted to personal preferences and be provided close to home (Patients, ICs, HCPs).” (p. 1)

“All groups stressed the importance of tailoring exercise to personal needs, possibilities and preferences. In general, this implied that exercise programs should not be ‘too demanding’ (Patients, ICs), and should be possible to do at home (Patients, ICs), or near home (Patients, ICs, HCPs).” (p. 4)

“They felt they knew best what was good for them, and wanted to determine for themselves how best to exercise, rather than follow instructions.” (p. 4)

“Most ICs (9/13) also preferred their loved one to exercise individually and at home but, in contrast to the patients, they believed exercise should be done under the guidance of a professional. This could be direct supervision, guidance via written materials (e.g., instructions, exercise examples) or by phone.” (p. 4)

Bruns, Argillander (112):

“In order to obtain compliance with a digitial prehabilitation device in this group, the introduction of a shared conversation at the beginning rather than a shared decision at the end was crucial.” (p. 14)

“With the aid of technology such as Fit4SurgeryTV, it is possible to easily adapt the program according to risk factors present and the patient’s own preferences.” (p. 15)

Singer, Soong (114):

“Further, participants found exercising at home while awaiting lung transplantation convenient.” (p. 7)

“[…] home-based prehabilitation program that leverages mobile health technology may be an inexpensive intervention not bound by geography or insurance that can safely and effectively improve frailty in lung transplant candidates.” (p. 10)

Agasi-Idenburg, Koning-van Zuilen (116):

“PA-Rehab should be individualized (FC) [*formal caregivers*].” (p. S61)

“Professionals prefer patients to exercise in groups with other patients, but patients prefer programs whereby they can exercise individually, near home, receiving professional advice only when needed; informal caregivers prefer that patients exercise individually but under direct professional supervision.” (p. S61)

Gill, Baker (119):

“PTEVAL [*computerized instrument*] made it possible for the therapists to closely monitor the progress of each participant and, in turn, likely contributed to the high completion rates for the specific interventions.” (p. 399)

“A prehabilitation program that is set in the home, rather than in a center, offers several potential advantages for frail older persons. First, there is a strong relation between the environment and function. Verbrugge and Jette have argued convincingly, for example, that disability is not a personal characteristic, but instead represents a gap between personal capacity and environmental demand. Setting the evaluation and intervention in the home allowed us to observe if participants could perform their mobility-related activities safely and effectively, given the demands of their living environment, and to act accordingly to modify the individual and/or the environment to ensure a safe and effective fit between capacity and demand.” (p. 398-399)

“[…] a home-based program was more appealing than a center-based program to many frail older persons, especially to those who do not have ready access to transportation and/or are reluctant to leave their homes. Many older persons, moreover, are familiar and comfortable with home-based services, given their proliferation in the past decade.” (p. 399)

Boreskie, Hay (121):

“[…] patient-centered approaches are needed to improve the feasibility and efficacy of prehabilitation. For example, access to prehabilitation should not be limited by barriers, such as transportation, which has beend reported as a common barrier to accessing rehabilitation programs. Creative solutions are needed to increase the accessibility to prehabilitation programs, such as the home-based prehabilitation pilot applied by Waite and colleagues or the home-based solution provided by Bruns and colleagues with their computer supported prehabilitation trial in patients awaiting colorectal cancer surgery.” (p. 580)

Lin, Visina (122):

“When patients are not able to achieve these goals, a supervised exercise approach at a rehabilitation facility is needed, although these programs are not available to most patients.” (p. 2114)

“Effective prehabilitation programmes must be individualised and pragmatic to address known barriers to adherence.” (p. 19)

“[…] an ideal exercise prehabilitation programme should thus be patient-centred and follow clinical best practices. It should be: (1) Geared towards maximum convenience and minimum burden by being home-based or colocated at a one-stop facility with other medical appointments. (2) Goal-directed, with individualised targets to incentivise and motivate patients. (3) Graded and progressive according to patients’ cardiovascular capacity.” (p. 21)

“The full value of prehabilitation can only be realised if high adherence rates are achieved. Individualised, goal-directed, pragmatic programmes which are sensitive to psychosocial factors and the underlying disease condition are most likely to succeed. Importantly, patients must be empowered and covinced of their value.” (p. 22)

“As prehabilitation is a programme where goals are personalised, its perceived benefits are necessarily individualised as well. For patients, subjective measures are thus an integral part of value analysis.” (p. 22)

Punt, van der Most (127):

„Die Kurz- und Langzeitziele sollten mit dem Patienten im Voraus besprochen werden (gemeinsame Trainingsziele).“ (p. 411)

„Die Planung, der Inhalt, die Struktur, die Durchführung und die Überwachung sollten in enger Zusammenarbeit mit dem Hochrisikopatienten und in seiner eigenen häuslichen Umgebung stattfinden.“ (p. 411)

„Das Ziel der Übungstherapie ist u.a. praktische Lösungen anzubieten, die von der Wohnsituation des Patienten abhängig sind. Beim Üben in häuslicher Umgebung werden patientenspezifische Probleme schneller entdeckt und Lösungen einfacher gefunden.“ (p. 411)

„Das Training sollte sowohl durch den Patienten als auch durch den Physiotherapeuten mithilfe von Funktionstests überwacht werden, damit die Dosis der Therapieeinheiten engmaschig kontrolliert und angepasst werden kann. Patienten sollten die Führung bei der Einschätzung des Therapieerfolgs und gegebenenfalls selbst die Initiative zur Übungssteigerung übernehmen, was eine höhere Motivation, eine Einhaltung des Therapieplans und Zufriedenheit zur Folge hat.“ (p. 411)

Wang, Stanforth (128):

“Taken together, the interventional features of this app highlight the potential of meeting individualized patient needs, providing motivation, enhancing patient engagement and adherence, and facilitating human interaction.” (p. 8)

Jensen, Lauridsen (130):

“[…] some patients might feel more secure in a supervised hospital-based exercise program while others may prefer to exercise independently in the comfort of their own homes. Both of these approaches have been implemented successfully; home-based training successfully results in improvements in patient functional status, although supervised exercise sessions appear to bring about more robust effects.” (p. 4)

“Recently, a greater emphasis has been placed on taking patient preference into consideration when designing prehabilitation programs. After all, if a patient does not enjoy or believe a program to be important, there is a greater chance that they will not participate. It is important to find out what the patient likes to do and how they like to do it when designing exercise programs so that adherence can be maximized, especially in a short time frame as in the presurgical period.” (p. 4)

“Providing psychological support in prehabilitation, with the degree of support reflecting the needs of each individual, may be seen as a means to promote recovery postsurgery but also to improve function and maximize program adherence in the presurgical phase.” (p. 5)

“Prehabilitation is not ‘one size fits all’ and it is essential that the nurse, as part of a multi-professional team, is aware of the importance of a comprehensive assessment to orchestrate a prehabilitation program that meets the needs of the individual and to motivate patient participation and adherence to the intervention.” (p. 6)

“Considering the relatively short window of opportunity for prehabilitation, it is critical to identify risk factors and understand the full physical and psychological needs of patients in order to prepare them best for surgery and recovery.” (p. 6)

Shovel and Morkane (131):

“Evidence suggests that positive behavior change can be engendered by setting exercise goals, identifying barriers to exercise, and keeping exercise diaries. Thus there is clear opportunity for individualised bespoke exercise regimens going forward.” (p. 649)

“The move to VC [*video conferencing*] has opened up prehabilitation to patients who have previously not been able to, or not wanted to, engage with the process. In our experience, cultural and language barriers, moderate to severe frailty, and excessive distance and travel were often responsible for preventing engagement with hospital-based in-person prehabilitation. We have seen such patients benefit from the move toward homebased prehabilitation. We observe that those who struggled with a language or cultural barrier are routinely now engaging with the support of famility within the home, and those who were previously disadvantaged by distance or travel owing to frailty are now on a level playing field.” (p. 650)

Waterland, Ismail (134):

“Prehabilitation should be administered in a robust manner that conforms to being structured and responsive to the individual patient, with an incremental increase in the frequency, intensity, and duration of exercise therapy.” (p. 897)

Carli, Bessissow (135):

“Particularly for the elderly one type of exercise does not fit all, and personalization of the exercise intervention is necessary and must be safe. In the case of prehabilitation, a structured program that specifies exercise intensity, frequency and modality is the goal.” (p. 322)

“All exercise interventions must be structured and personalized as one exercise does not fit all patients.” (p. 324)

“If patient is not able to perform exercises alone at home, it is recommended to offer at least one supervised session per week to make sure the exercises are executed properly. Such approach provides patients’ confidence that the exercises can be continued to be executed at home, and improves compliance to the training.” (p. 324)

Oosting, Jans (136):

“[…] preoperative exercise should focus on frail elderly patients and should be functional, targeting their limitations in activities and participation and taking into account environmental and personal factors.” (p. 611)

“We found that the best way to monitor functional capacity of each individual patient was using a tailor-made functional circuit standardized for each individual.” (p. 614)

“[…] point out the relevance of personal and environmental factors like habit strength, coping, and the attitude of caregivers and family. We took into account some of these factors like exercising at home, involving family, and encouraging active coping.” (p. 615-616)

Williams, Berzigotti (138):

“Supervised programs provide structure, camaraderie and access to regular health care professional support, yet can be difficult for patients to adhere to due to cost/time of travel and fluctuating health status. Unsupervised, home-based exercise provides flexibility, limits patient travel and promotes independent lifestyle changes in the patient’s own environment.” (p.995)

Yau, Underwood (139):

“Good mobility status and closer residential distance to the hospital were associated with successful RCT participation. Recruitment strategies that address ambulatory and transport difficulties may increase the participation of patients living far away from the hospital.” (p. 19)

Afilalo (142):

“If difficulties with transportation and mobility are elicited as major barriers to a attend center-based facility, home-based CR should be considered as a viable alternative.” (p. 449)

Heil, Driessen (143):

“Combining appointments on a single day” (p. 6)

“Incorporate social environment to facilitate patient with prehabilitation program” (p. 7)

“Implementation of digital tools for interaction and reduction of travel distance” (p. 7)

“Offering an intervention program close to home” (p. 8)

“Personal support during prehabilitation program” (p. 8)

“Set goals and motivate patients to accomplish them” (p. 8)

“Identified facilitators for the practice environment included combining patient appointments as it would not only lead to a decrease in the number of hospital visits for patients but could also ensure accessible contact between involved healthcare professionals. In addition, offering an intervention program close to home and implementation of digital tools were suggested options to reduce travel distances and facilitate patients’ compliance.” (p. 9)

“Patient’s gaining insights in their movement patterns and being able to set personal goals as well as including their social environment could all potentially facilitate adoption by patients. Also, group activities where patients would be able to exchange experiences and motivate peers were identified as a facilitating factor.” (p. 11)

“Important facilitators were the ability to offer a personalized prehabilitation program for each individual.” (p. 11)

“To implement an innovation such as prehabilitation in clinical practice, an individualized program with regard to content, duration, and setting is needed. In order to create more patient-centeredness, questions including what, when, where, who, and why should be taken into account while developing future prehabilitation programs.” (p. 11)

“Previous studies, interviewing patients, highlighted already the importance of appropriate information provision and an accessible personalized prehabilitation program” (p. 12)

Yau and Tsang (144)

“Holistic prehabilitation programmes should be multimodal and ideally target not only the biological, but also the psychological and social risk factors that hinder the recovery after the huge stress brought by cardiac surgery.” (p. 16-17)

**Guidance and (social support)**

Singer, Soong (113):

“Qualitatively, participants report the ‘app’ platform is easy to use and that the embedded gamification features and Fitbit trackers help in motivation.”

Agasi-Idenburg, Zuilen (117):

“All groups mentioned the importance of social support. Patients and ICs [*informal caregivers*], in particular, emphasized how important it is to receive/give practical and emotional support in relation to exercising.” (p. 4)

“[…] it was acknowledged that exercising in groups might stimulate adherence to the program” (p. 4)

Bruns, Argillander (112):

“Twelve patients (86%) regarded the reward after the operation as an additional motivation.” (p. 12)

Lin, Visina (122):

“Remote monitoring of on-training adherence may be the best approach to home-based prehabilitation and can be achieved with affordable personal activity trackers.” (p. 2114)

Perlmutter, Ali (124):

“[…] more consistent guidance and active engagement with patients as they complete a prehabilitation regimen is crucial for consistent participation, accurate data collection and maximizing potential postoperative benefits.” (p. 715)

Wang, Stanforth (128):

“Social support (e.g., support from friends, family members, and neighbors) is another key construct for enhancing adherence to exercise interventions among patients with cancer as well as a critical element in home-based interventions and self-management mHealth app-based interventions.” (p. 8)

Shovel and Morkane (131):

“Evidence suggests that positive behavior change can be engendered by setting exercise goals […]” (p. 649)

Rumer, Saraswathula (132):

“An exciting new arena is using WFT [wearable fitness trackers] to help patients and providers monitor patients’ health and deliver health interventions.” (p. 188)

“Many devices have social support functions that can be used to target patient motivation and adherence. These devices may be the next frontier in prehabilitation to improve outcomes of frail patients undergoing major surgery such as transplant.” (p. 189)

“Newer commercially available WFTs offer online communities to share progress and compete with friends and relatives. Goals of social support are to increase motivation and adherence with exercise programs. Patient motivation is a major factor in determining the success of prehabilitation programs, and the utilization of social networks with WFTs could represent a new way to attack the issue of patient motivation and adherence.” (p. 192)

Bongers, Klaase (133):

“[…] frequent monitoring of training progress is important to motivate responders, to timely identify nonresponders, and to subsequently make necessary program adjustments concerning trainin frequency, intensity, and duration.” (p. 896)

Hoogeboom, Dronkers (137):

“To facilitate adherence to the programme we monitored progression, let patients include their spouse/family in the exercises, implemented feedback moments about the intensity of and attitude towards the exercise, and progressed training parameters gradually.” (p. 903)

Williams, Berzigotti (138):

“[…] regular contact with a health care professional is likely needed to promote adherence, with specific focus on patients’ motivations and psycho-behavioural barriers towards exercise.” (p. 995)

Heil, Driessen (143):

“Group activities to exchange experiences and motivate peers” (p. 7)

“Set goals and motivate patients to accomplish them” (p. 8)

“Also, group activities where patients would be able to exchange experiences were identified as a facilitating factor.” (p. 11)

**Integration into the setting**

Agasi-Idenburg, Zuilen (117):

“[…] low cost program (ICs, HCPs) facilitate exercise.” (p. 1)

Gill, Baker (119):

“Many older persons, moreover, are familiar and comfortable with home-based services, given their proliferation in the past decade. Finally, if found to be cost effective, a home-based program could be easily incorporated into the array of services offered by home-care agencies.” (p. 399)

Arora, Brown (129):

“The interaction of key factors such as stakeholders’ perception regarding the quality of evidence, the organizations’ contextual readiness, the facilitation to the integration process, and the postimplementation process evaluation determines the success of an evidence-based practice intervention implementation process.” (p. 845)

“The contextual readiness in terms of the leadership support, the flexibility of existing surgical practice culture, data processing capabilities and availability of resources, and decision-making authority to implement prehab are critical aspects of an implementation-ready organization.” (p. 845)

Bongers, Klaase (133):

“To maximize participation rate, adherence, and effectiveness in high-risk patients, a preoperative exercise program must be integrated in the perioperative trajectory and performed in the patient’s preexistent living context, thereby (partly) supervised by a competent community physical therapist and involving the patient’s informal support system.” (p. 896)

Carli, Bessissow (135):

“The preoperative clinic represents an ideal set up for such a comprehensive program, where all the experts like anesthesiologists, internists, surgeons, physiotherapists, nutritionists, nurses, psychologists can convene and define the most appropriate for surgical preparation of complex cases. When needed geriatricians, psychiatrists, oncologists need to be consulted.” (p. 323)

Williams, Berzigotti (138):

“[…] costly equipment may not be required to produce positive improvements in aerobic capacity and function for patients with CLD [*chronic* *liver disease*].” (p. 993)

Heil, Driessen (143):

“Application of prehabilitation fits in hospital strategy” (p. 6)

“Include skeptical healthcare professionals in prehabilitation team from adoption phase” (p. 7)

“Introduce prehabilitation as part of regular care” (p. 7)

“Introduce prehabilitation early in trajectory” (p. 7)

“Including skeptic healthcare professionals early in the adoption phase of the innovation could facilitate and overcome this [*unawareness of importance of prehabilitation*].” (p. 9)

“In the monitoring phase, an ambassador, who could persuade, enthuse, and unite coworkers, is necessary for the diffusion, dissemination, and implementation of prehabilitation in the hospital for the long term.” (p. 11)

“For transferring prehabilitation within the practice environment, an ambassador was deemed as an important facilitator.” (p. 11)

“[…] implementation of prehabilitation requires adjustments in the hospital as practice environment. Local adjustments in the organization of preoperative colorectal cancer care pathways are needed to create availability of dedicated resources and time for involved healthcare professionals. The presence of a program coordinator, for example an oncology nurse, can facilitate effective implementation. This program coordinator can overview the program and signals arising problems on both organizational and patient level.” (p. 11)

“[…] it is important that adopters in the preadoption stage are aware of the innovation.” (p. 11-12)

“skeptics in the surgical pathway need to be included in the prehabilitation team and early in the adoption phase to convince them of the potential merits of prehabilitation and to ensure appropriate information provision towards patients.” (p. 12)

Gurlit and Gogol (123):

“Prehabilitation appears particularly promising when administered within the scope of multidisciplinary collaboration and as an integrated concept including prehabilitation, perioperative intervention and rehabilitation. With preoperative risk evaluation being an essential requirement for tailored anaesthesia and appropriate surgical treatment, the anaesthesiologist should occupy a key position in further distribution of prehabilitation.” (p. 112)

**Multidisciplinary team approach**

Gurlit and Gogol (123):

“[…] aiming at real advantages for this vulnerable group prehabilitation in geriatric patients can succeed only when understood as an appeal to cooperation between all professions involved.” (p. 112)

“[…] beyond dispute is the essential requirement of interdisciplinary teamwork if we are to succeed in surgical treatment in old an frail patients.” (p. 113)

Ng, Lee (125):

“a fragmented approach fails to address the holistic needs of the older surgical patient, particularly in the presence of frailty and multimorbidity. Multimodal prehabilitation is the antidote to this fragmentation, as the three modalities [*exercise, nutrition, psychological preparation*] are in fact interdependent.” (p. 20)

“We believe the elements of exercise, nutrition and patient engagement are necessary and synergistic, and their delivery as a package by a multidisciplinary team holds great potential for improvement of surgical outcomes.” (p.20)

Jensen, Lauridsen (130):

“[…] the nurse is an important key-player in a successful implementation of these programs in clinical practice and coordinator of the various elements that will benefit the patient.” (p. 6)

“Multimodal prehabilitation requires: a willingness to change practice, strong anchor-based leadership and a multi-professional approach, as it proposes a shift from the current health care paradigm.” (p. 6)

Shovel and Morkane (131):

“These factors include strong leadership and multidisciplinary working teams, engaged stakeholders, senior managerial and executive support, and dedicated funding and resources.” (p. 649)

“It involves coming together and dedication of major stakeholders and team members: vascular surgeons, vascular nurse specialists, physiotherapists and exercise therapists, dieticians, occupational therapists, and anaesthesists, to name but a few. Each of these specialists play an active role in delivering the programme reliably and successfully.” (p.650)

Carli, Bessissow (135):

“Such programs requires a paradigm shift in the sense that all players need to be working in team for the benefit of the patients’s outcome.” (p. 321)

“The main goal of prehabilitation is therefore to improve the clinical, physical, nutritional and emotional aspects of patient wellbeing. All these elements when put together have a greater and synergistic effect than each element administered alone. Such approach then requires a multidisciplinary team who are able to interact together and provide a personalized, structured protocol.” (p. 322)

Heil, Driessen (143):

“Accessible contact between involved healthcare professionals” (p. 6)

“An ambassador should persuade, enthuse, and unite coworkers” (p. 6)

“Coordination of program and program appointments by a specialized nurse” (p. 6)

“Delay surgery if necessary” (p. 6)

“Preoperative multidisciplinary prehabilitation consultation” (p. 8)

“Although the solution of an additional is considered time-consuming, it was thought of as enhancing program sustainability and team building.” (p. 9)

“Identified facilitators for the practice environment included combining patient appointments as it would not only lead to a decrease in the number of hospital visits for patients but could also ensure accessible contact between involved healthcare professionals.” (p. 9)

“Contact through multidisciplinary consultation in order to identify eligible patients and monitor a patients’ progress was identified as facilitator. To partially overcome the problem of time-consuming extra multidisciplinary consultations, it was stated that evaluation of individual patients may only be necessary in case of problems or deviations from the program.” (p. 9)

“[…] it should be possible to delay the procedure if deemed necessary due to patient’s performance status.” (p. 9)

“[…] physicians’ leadership is essential to facilitate diffusion, dissemination, and implementation of prehabilitation both on micro (clinical integration), meso- (professional and organizational integration) and macro- (system integration) levels” (p. 12)

Yau and Tsang (144):

“Successful implementation of prehabilitation interventions requires multidisciplinary collaboration, specifically including surgeons, anaesthesiologists, intensivists, physiotherapists, dieticians, psychologists and nurses, in order to address individual needs of the patients holistically.” (p. 16)

**Clear patient pathway**

Lin, Visina (122):

“Early referral to prehabilitation is encouraged, so patients may engage in exercise and execute home-based training before physical decline worsens.” (p. 2114)

Gurlit and Gogol (123):

“[…] with the identification of patients at risk only around the time of surgery, anaesthesiologist and patient at risk do not meet at the most opportune time to intervene for the purpose of increasing the patient’s functional reserve. Thus, once the decision is for elective surgery is taken, the patient should meet the anaesthesiologist where the evaluation of possible risk factors and introduction of corresponding measures can immediately be started with an adequate time interval long enough for significant improvements.” (p. 112)

“Prehabilitation should not be understood as a single concept but must be seen as an essential part of the whole care process in geriatric patients. As some colleagues still do not agree on this, we strongly believe that prehabilitation must be seen as a crucial element in an integrative pathway, which we call SAPIF2 [Screen and Assess, Prehabilitation, Intervention, Follow-up (short-term) and Follow-up (long-term)].” (p. 112)

Carli, Bessissow (135)

“It is especially helpful to refer the patient at the first visit so that the timeline to surgical or other care can be respected without undue delays.” (p. 324)

Heil, Driessen (143):

“Adjust patient selection during implementation based on (local) setting” (p. 6)

“At last, prehabilitation should be introduced early in the diagnostic trajectory to create sufficient time for prehabilitation while still meeting the national guidelines for timely treatment after diagnosis.” (p. 9)

“Another adjustment in the organization of preoperative colorectal cancer care should be the possibility to lengthen the time interval between operation indication and surgery, which could serve as a protective time interval to battle negative oncological outcomes. As the mandatory standards and operation room planning currently determine the time between indication and actual surgery, it should rather the surgeon determining (extended) time until surgery based on the patients’ physical condition and nutritional status and the ability to improve this by prehabilitation.” (p. 11)

**Barriers:**

**Overwhelming Information Provision**

(112):

“The fact that an operation may have to be delayed in order to complete a training program poses a mental challenge for many patients as a longer waiting period may be perceived to promote tumor growth and increase the risk of metastases.” (p. 14)

Agasi-Idenburg, Zuilen (117)

“[…] not receiving or misunderstanding information […] are barriers to preoperative exercise.” (p. 2)

“With regard to how information on exercise should be given, some participants said it should be primarily in writing, because they could not remember verbal instructions. Other participants (patients, ICs) mentioned that written information could be overwhelming, because there was so much to read, or because the information itself would upset patients. HCPs mentioned that information was often not read and, if it was read, it was not always understood.” (p. 5)

“[…] with many appointments at the hospital that are planned for them, and they seem to lose their direction. It takes time to adjust to such a situation, and it likely takes even more time to appreciate the importance of and actually pursue exercise during this critical period.” (p. 5)

“Patients need to be informed about the importance of improving their physical condition before surgery, preferably by a physician. However, it appears that advice on exercise if often not given, not heard, or not understood.” (p. 5)

Mohamed, Ramachandran (126):

“In a feasibility study of home-based cognitive prehabilitation in older surgical patients, barriers to cognitive training included feeling overwhelmed, technical issues with training, and the time commitment.” (p. 8)

Shovel and Morkane (131):

“Reliance on the patient ‘to do their bit’ can pose an interesting and at times difficult challenge to prehabilitation teams.” (p. 651)

Oosting, Jans (136):

“Although we advised patients to take the pain medications prescribed by their doctors, most patients did not.” (p. 614)

Furyk, Senthuran (141):

“Timing of recruitment: Patients were approached in a busy surgical clinic immediately after receiving the news that they had colorectal cancer and required major surgery. Many patients, understandably, were minimally able to process further information, and preferred not to consider involvement in research at that time.” (p. 3)

Afilalo (142):

“[…] their own lack of perceived benefit or motivation when not adequately informed about the importance of CR [*cardiac rehabilitation*] as a treatment for their underlying heart disease.” (p. 449)

Heil, Driessen (143):

“The idea that sedentary behavior is necessary when cancer is diagnosed” (p. 5)

“The idea that tumor should be removed as soon as possible” (p. 5)

“With regard to patients, the dominating ideas about illness behavior were detrimental as they often believe that sedentary behavior is necessary when cancer is diagnosed. Also, patients believe that the tumor needs to be removed as soon as possible after diagnosis.” (p. 9)

“[…] incorrect ideas of patients about what is important in the preoperative phase were mentioned as serious barriers.” (p. 11)

“If potential benefits of prehabilitation remain unclear for recipients, transforming care towards more integration is difficult, and consequently, demonstration of efficacy will fail due to low program adherence. Physicians in particular are the principal players to break this vicious cycle by either supporting or opposing successful transformative efforts.” (p. 12)

**Lack of Multi-Modality and/or Adaptability**

Feng, Dorrance (111):

“[…] participants identified potential improvements to the program: more individualized, a variety of resistance bands of different strengths, and more exercise equipment.” (p. 180)

Bruns, Argillander (112):

“Offering all patients a one-size-fits-all intervention fails to take individual preferences into account which can lead to low compliance.” (p. 13)

Gill, Baker (119):

“[…] home-based programs may offer a lower training stimulus, partly because there is no direct supervision during most of the exercise sessions.” (p. 399)

McAdams-DeMarco, Ying (120):

“[…] top three reasons for refusing to participate were (a) geographical limitations (clinic is too far or not conveniently located; unable to commute); (b) too much commitment (weekly appointments difficult to fit into schedule because of work, school, medical or dialysis appointments; too much of a time commitment); and (c) transportation issues (dependent on relative or someone for transportation).” (p. 4)

“[…] reasons provided for not attending prehabilitation sessions included transportation issues, schedule conflicts, health/medical issues, and too much of a time commitment.” (p. 4)

“While feasible, our intervention was limited in that it was challenging to enroll participants who lived further from the center.” (p. 6)

Lin, Visina (122):

“Limitations to successful prehabilitation include the inability to provide tailored exercise recommendations during hepatology clinic visits, […] and burden generated on patients and their caregivers (i.e., supervision, transportation, training fees).” (p. 2106)

“Although PT strived to tailor the exercise prescription to varying individual needs, physical and logistic barriers remained, such as symptoms of fatigue, unplanned hospitalizations, lack of interest, and failure to recognize the benefits of exercise.” (p. 2115)

Punt, van der Most (127):

„Ältere fragilere Patienten neigen eher dazu, an „home-based“ als an „clinic-based“ Therapien teilzunehmen. Bei diesen Patienten könne schließlich die Anreise zur Klinik/Praxis erhebliche Probleme (Reisedistanz, Treppe, Hilfsmittel) verursachen und dafür sorgen, dass bei der Ankunft nicht mehr ausreichend Energie für die Übungstherapie übrig bleibt.“ (p. 411)

Wang, Stanforth (128):

“Even when resistance training exercises are included, there is often low adherence to such a program owing to its challenging nature, even though resistance training is crucial for improving muscle strength and will often predict a lower incidence of postoperative complications. Additionally, the largest barrier to participating in prehabilitation reported by patients has been a lack of transportation (i.e., arranging transportation and finding or paying for parking) and convenience making any attempt at effective supervision of a multidisciplinary program much more difficult.” (p. 2)

Shovel and Morkane (131):

“[…] multiple patient reported barriers, including lack of tie, illness and fatigue, not being interested, and safety concerns.” (p. 649)

“Logistical Challenges” (p. 649)

Oosting, Jans (136):

“[…] more than 60% of the participants, especially the most frail patients, had problems participating because of the distance they had to travel to the outpatient department where the training was given and because they did not have enough energy to perform the exercises.” (p. 611)

“Some patients were limited in their ability to exercise outdoors because of heavy snowfall.” (p. 612)

“Monitoring patients and finding the right intensity level was a matter of trial and error.” (p. 614)

Hoogeboom, Dronkers (137):

“[…] eligible patients did not participate, mainly because of logistic considerations.” (p. 901)

“[…] patients declined (main reasons: no transport (n = 17), no energy (n =10), no time (n = 6), not motivated (n = 4), and some already had therapy (n = 4)).” (p. 904)

“[…] most patients were unable to drive a car and were dependent on friends or family to take them to the hospital twice a week.” (p. 906)

“Most frail patients were unable to participate in additional exercise therapy provided at an outpatient physiotherapy department, as a result of logistic considerations.” (p. 908)

Williams, Berzigotti (138):

“Patients with CLD [*chronic liver disease*] notoriously have lower exercise tolerance levels and often fatigue early, making them unlikely to push themselves without professional guidance.” (p. 991)

Furyk, Senthuran (141):

“[…] barriers were: shorter than expected operative times […]” (p. 3)

“[…] ability of frail elderly to surmount logistical problems was limited, given their reduced independence with transport and potential memory problems.” (p. 4)

Durand, Beckert (140):

"[…] limitations such as compliance, lack of capacity to exercise, […], and time required for effect are all significant limitations.” (p. 384)

Afilalo (142):

“The barriers to CR [*cardiac rehabilitation*] participation in frail patients are cultural and contextual nature. Clinicians and researchers frequently dismiss frail patients as ‘too unfit for CR’ or ‘inappropriate (for CR) due to frailty’, concerned about their inability to complete a standard exercise program, their risk of adverse events, and a lack of perceived benefit given the paucity of evidence in this demographic.” (p. 449)

“Patients are concerned about their competing priorities for time- and cost-intensive health care visits, their transportation to CR appointments multiple times per week […]” (p. 449)

“If difficulties with transportation or mobility are elicited as major barriers to attend a center-based facility, home-based CR should be considered a viable alternative.” (p. 449)

Heil, Driessen (143):

“Combining appointments is difficult due to different work activities” (p. 10)

“Patients are unable to visit hospital frequently” (p. 10)

“Previous qualitative studies already highlighted […] key barriers: […] inconsistent practice and poor patient engagement.” (p. 2)

“[…] perceived complexity of prehabilitation and differences in patients’ resilience and training opportunities (i.e., a ‘one size fits all’ prehabilitation program would not work) was seen as a barrier.” (p. 9)

“Some patients were not capable to visit the hospital frequently, while combining prehabilitation appointments with different healthcare professionals was also considered to be difficult because of different work activities of involved healthcare professionals.” (p. 9)

“[…] the timing of surgery was identified as a logistic problem. The inflexible and rapidly changing operation room planning would often take priority over the prehabilitation program, resulting in an early termination of the prehabilitation program. At the same time, national quality indicators state that treatment should take place within 6 weeks of diagnosis, making the time window for prehabilitation often (too) short.” (p. 9)

“Important barriers included […] the inability of patients to follow a predefined hospital-based prehabilitation program (due to a lack of personalized programs or inflexibility of ‘prescribed’ prehabilitation)” (p. 11)

**Fragmentation and Misalignment of Providers**

Agasi-Idenburg, Zuilen (117):

“An important environmental barrier to preoperative exercise mentioned by all three groups of participants was the many hospital appointments patients had to attend. This made scheduling of regular exercise difficult.” (p. 4)

Johanning and Hall (118):

“[…] realizing the full potential of SWPs [*surgical wellness programs*] will require a shift in philosophy on the part of both surgeons and their patients. Prehabilitation often requires a sudden and significant lifestyle change and personal value assessment that may be challenging even in the best of circumstances. On the part of surgeons, the initial decision to operate was made before SWP referral, putting into motion a chain of events that is not easily altered even with a comprehensive and multidisciplinary risk assessment that suggests that different treatment options may be better aligned with patient goals. The shifts necessary for SWP adoption with a goals-of-care focus will require additional training and incentives understanding such cultural change will likely be slow.” (p. 1)

Lin, Visina (122):

“Limitation to successful prehabilitation include […] limited experience with ESLD [*end-stage liver disease*] among exercise professionals […]” (p. 2106)

Ng, Lee (125):

“In the absence of full endorsement by attending surgeons, downstream effects on prehabilitation interventions include miscommunication and misaligned goals among the healthcare team and lack of commitment on the part of patients. The end result is resource wastage and suboptimal outcomes.” (p. 21)

Mohamed, Ramachandran (126):

“Likewise, surgeons may object to the added complexities of scheduling introduced by prehabilitation. A clear conception of the duration of a prehabilitation program may alleviate this concern.” (p. 9)

Arora, Brown (129):

“In particular, the target populations who might derive the most benefit from prehab are not well defined.” (p. 843)

Heil, Driessen (143):

“Healthcare professionals are unaware of (importance of) prehabilitation programs” (p. 4)

“Operating room planning takes precedence over prehabilitation program” (p. 4)

“Previous qualitative studies already highlighted […] key barriers for healthcare professionals to implementing a prehabilitation program: knowledge, […], inconsistent practice […]” (p. 2)

“The lack of structural program implementation evaluation in a team meeting to identify and resolve experienced problems was mentioned as well.” (p. 9)

“[…] unawareness of the importance and possibilities of a prehabilitation program by healthcare professionals was an important barrier.” (p. 9)

“Besides, unawareness of (importance of) the prehabilitation program among both healthcare professionals and patients […] were mentioned as serious barriers.” (p. 11)

**Resource Constraints**

Agasi-Idenburg, Zuilen (117)

“[…] the time available between diagnosis and surgery is typically quite short, reflecting the pressure to minimize waiting time as an indicator of quality.” (p. 5)

Johanning and Hall (118)

“[…] logistical challenges of serving the ‘Silver Tsunami’ will require a solution that can be expanded.” (p. 1)

“If all suitable individuals were referred to and evaluated by the SWP for 90-minute visits and 4 to 6 postvisit coaching calls, the clinical demand could outstrip existing resources, both human and financial.” (p. 1)

“[…] a sustainable business model for SWPs remains uncertain.” (p. 1)

Lin, Visina (122):

“Limitations to successful prehabilitation include […] lack of reimbursement […]” (p. 2106)

Ng, Lee (125):

“Multimodal prehabilitation can be resource-intensive. It is thus rational to target the intervention at patients with the greatest potential to benefit.” (p. 20)

Mohamed, Ramachandran (126):

“A second barrier to prehabilitation may be the resources required for its implementation. Resources are different for a daily supervised exercise regimen supported by CBT and nutritional education compared with a home-based program supported through an activity monitor, reminder phone calls, and limited in-person instruction.” (p. 9)

Arora, Brown (129):

“[…] prehab requires a substantial commitment from the patient as well as resources from the health care team.” (p. 843)

Shovel and Morkane (131):

“[…] at one centre, we are unable to offer psychologic prehabilitation and offer only dietetic and exercise prehabilitation. Yet at our other 2 centres, only a few kilometres away, we offer a trimodal model of exercise, dietetic, and psychologic prehabilitation.” (p. 646)

“[…] prehabilitation remains a resource-intensive intervention. And this can become a challenge for both aspiring and existing prehabilitation programs alike.” (p. 650)

“[…] prehabilitation is a resource-heavy and time-intensive intervention, and ‘getting it right’ is crucial.” (p. 651)

Williams, Berzigotti (138):

“Over half (64%) of the aerobic exercise interventions utilized expensive equipments such as treadmills and cycle ergometers. Although this help guides training intensity and progression, the widespread applicability of such approach is limited, especially in health services with restricted resources. Moreover, accessibility to such equipment for patients beyond the realms of a research study may be limited, thus creating barriers to long-term lifestyle and exercise changes for patients with CLD [*chronic liver disease*].” (p. 991)

Durand, Beckert (140):

“[…] limitations such as […] cost […] are all significant limitations.” (p. 384)

“Supervised exercise may be a solution to the low compliance; however, this strategy may not be feasible due to cost, adequate staffing, and overall inconvenience for the patient.” (p. 383)

Heil, Driessen (143):

“Costs must be financed immediately while yields are not (directly) clear” (p. 4)

“Counseling patients is time consuming” (p. 4)

“Healthcare system is not adapted, including availability of paramedics in hospital” (p. 4)

“Indirect costs for patients (e.g. travel expenses)” (p. 4)

“Multidisciplinary consultation is time consuming” (p. 4)

“Previous qualitative studies already highlighted […] key barriers for healthcare professionals to implementing a prehabilitation program: […] resource […].” (p. 2)

“Especially in combination with (high) immediate costs and no directly measurable or visible yields, it was often concluded that advantages of prehabilitation were unclear.” (p. 9)

“Also, the combination of counseling patients for prehabilitation and an additional multidisciplinary consultation was seen as time-consuming.” (p. 9)

Boreskie, Hay (121):

“The opportunity to intervene preoperatively is restricted by the length of preoperative period. Waitlists and urgency of the procedure can impact this length of time.” (p. 573)

Yau and Tsang (144):

“[…] the opportunity for prehabilitation and the duration of prehabilitation training were dependent greatly on the length of period before surgery, which was on occasion restricted by the waitlist, and urgency of procedures.” (p. 136)

“[…] the criteria on waiting time was not adopted after 1^st^ April 2019 since there were large variations in the surgical waiting time experience in real clinical situation.” (p. 91)

**Lack of (Social) Support**

Ng, Lee (125):

“[…] a significant barrier to referral was identified as lack of physician support, attributed to a lack of conviction regarding the benefit of prehabilitation, especially when weighed against potential safety concerns of delayed treatment.” (p. 21)

Furyk, Senthuran (141):

“Patients felt that they were a burden on family and friends, especially for transport.” (p. 3)

1. Hirschhorn AD, Kolt GS, Brooks AJ. Barriers and enablers to the provision and receipt of preoperative pelvic floor muscle training for men having radical prostatectomy: a qualitative study. BMC health services research. 2013;13(1):1-14.

2. Francis-Coad J, Edgar D, Bulsara CE, Barrett-Lennard A, Owen K, Fletcher D, et al. Partnering with patients to design a prehabilitation program for optimizing the patient experience through general surgery. Patient Experience Journal. 2021;8(1):135-47.

3. Brown OH, Kenney BC, Derstine BA, Grenda DR, Sullivan JA, Palazzolo WC, et al. Patient Engagement Drives the Positive Impact of Prehabilitation. Michigan Journal of Medicine. 2018;3(1).

4. Boden I, El-Ansary D, Zalucki N, Robertson IK, Browning L, Skinner EH, et al. Physiotherapy education and training prior to upper abdominal surgery is memorable and has high treatment fidelity: a nested mixed-methods randomised-controlled study. Physiotherapy. 2018;104(2):194-202.

5. Grimmett C, Bradbury K, Dalton SO, Fecher-Jones I, Hoedjes M, Varkonyi-Sepp J, et al. The role of behavioral science in personalized multimodal prehabilitation in cancer. Frontiers in Psychology. 2021;12:261.

6. Davis JF, Van Rooijen SJ, Grimmett C, West MA, Campbell AM, Awasthi R, et al. From Theory to Practice: An International Approach to Establishing Prehabilitation Programmes. Current Anesthesiology Reports. 2022:1-9.

7. Beck A, Vind Thaysen H, Hasselholt Soegaard C, Blaakaer J, Seibaek L. Prehabilitation in cancer care: patients’ ability to prepare for major abdominal surgery. Scandinavian journal of caring sciences. 2021;35(1):143-55.

8. Boote J, Newsome R, Reddington M, Cole A, Dimairo M. Physiotherapy for patients with sciatica awaiting lumbar micro‐discectomy surgery: A nested, qualitative study of patients' views and experiences. Physiotherapy Research International. 2017;22(3):e1665.

9. Bates A, West M, Jack S. Framework for prehabilitation services. Journal of British Surgery. 2020;107(2):e11-e4.

10. Husted RS, Bandholm T, Rathleff MS, Troelsen A, Kirk J. Perceived facilitators and barriers among physical therapists and orthopedic surgeons to pre-operative home-based exercise with one exercise-only in patients eligible for knee replacement: A qualitative interview study nested in the QUADX-1 trial. PLOS ONE. 2020;15(10):e0241175.

11. McLaughlin J, Palmer C, Redwood S, Kipping R, Owens L, Reynolds R, et al. Commissioner, clinician, and patient experiences of a pre‐surgical health optimisation programme–a qualitative study. BMC Health Services Research. 2021;21(1):1-10.

12. Zhu H, Moffa Z, Wang X, Abdullah S, Julaiti J, Carroll J, editors. Understanding challenges in prehabilitation for patients with multiple chronic conditions. Proceedings of the 12th EAI International Conference on Pervasive Computing Technologies for Healthcare; 2018.

13. Ismail H, Cormie P, Burbury K, Waterland J, Denehy L, Riedel B. Prehabilitation prior to major cancer surgery: training for surgery to optimize physiologic reserve to reduce postoperative complications. Current Anesthesiology Reports. 2018;8(4):375-85.

14. Shukla A, Granger CL, Wright GM, Edbrooke L, Denehy L. Attitudes and perceptions to prehabilitation in lung cancer. Integrative cancer therapies. 2020;19:1534735420924466.

15. Drummond K, Lambert G, Tahasildar B, Carli F. Successes and challenges of implementing teleprehabilitation for onco-surgical candidates and patients’ experience: a retrospective pilot-cohort study. Scientific Reports. 2022;12(1):6775.

16. Norris CM, Close JC. Prehabilitation for the frailty syndrome: improving outcomes for our most vulnerable patients. Anesthesia & Analgesia. 2019;130(6):1524-33.

17. Catho H, Guigard S, Toffart A-C, Frey G, Chollier T, Brichon P-Y, et al. What are the barriers to the completion of a home-based rehabilitation programme for patients awaiting surgery for lung cancer: a prospective observational study. BMJ Open. 2021;11(2):e041907.

18. Moore J, Scoggins CR, Philips P, Egger M, Tennant P, Little J, et al. Implementation of prehabilitation for major abdominal surgery and head and neck surgery: a simplified seven-day protocol. Journal of Gastrointestinal Surgery. 2021;25(8):2076-82.

19. Abdelmasih M. Prehabilitation Prior to Surgery: A Scoping Review and Pilot Quality Improvement Project [M.Sc.]. Ann Arbor: University of Toronto (Canada); 2021.

20. Anonymous. Response to "Prehabilitation for Patients With Esophageal Cancer During Neoadjuvant Treatment and Surgery". Journal of Surgical Research. 2019;242:3.

21. Chen H, Ferrando AA, White MG, Pauly M, Bartter T, Dunn MA, et al. A randomized pilot trial of home-based physical activity plus dietary intervention to improve physical function in patients with advanced liver disease. American Journal of Transplantation. 2019;19(Supplement 3):717-8.

22. Fromm CG, Metzler DJ. Preparing your older patient for surgery. Registered Nurse. 1993;56(1):38-42.

23. Gillis C, Ljungqvist O, Carli F. Prehabilitation, enhanced recovery after surgery, or both? A narrative review. British Journal of Anaesthesia. 2022;128(3):434-48.

24. Halliday LJ, Doganay E, Wynter-Blyth VA, Hanna GB, Moorthy K. The Impact of Prehabilitation on Post-operative Outcomes in Oesophageal Cancer Surgery: a Propensity Score Matched Comparison. Journal of Gastrointestinal Surgery. 2021;25(11):2733-41.

25. Horattas I, Visioni A, Horattas MC. Improving Surgical Outcomes Prior to Incision: Superior Outcomes in the Geriatric Surgery Population after Prehabilitation. Journal of the American College of Surgeons. 2020;231(4 Supplement 2):e145.

26. Kaye DR, Thelen-Perry S, Schafer C, Qin Y, Reger HI, Parker C, et al. The feasibility, safety and impact of a prehabilitation program for patients undergoing cystectomy. Journal of Urology. 2019;201(4):e926‐e7.

27. Palmer J, Pymer S, Smith GE, Harwood AE, Ingle L, Huang C, et al. Presurgery exercise-based conditioning interventions (prehabilitation) in adults undergoing lower limb surgery for peripheral arterial disease (Cochrane review) [with consumer summary]. Cochrane Database of Systematic Reviews 2020;Issue 9. 2020.

28. Pang NQ, Tan YX, Samuel M, Tan KK, Bonney GK, Yi H, et al. Multimodal prehabilitation in older adults before major abdominal surgery: a systematic review and meta-analysis. Langenbeck's Archives of Surgery. 2022.

29. Rapti A, Kerenidi T, Zarogoulidis K. Treatment of lung cancer. Pneumon. 2012;25(SUPPL.1):60-6.

30. Santa Mina D, Alibhai SMH. Prehabilitation in geriatric oncology. Journal of Geriatric Oncology. 2020;11(4):731-4.

31. Slim K, Selvy M, Theissen A. Prehabilitation before major surgery: growing interest but persistent haze. Anaesthesia Critical Care and Pain Medicine. 2021;40(2) (no pagination).

32. Wynne S, Dickinson F, Fraser S, Peat N, Labuc P, Bracegirdle R, et al. OA08.04 Providing Thoracic Prehabilitation during COVID-19: Review of a Virtual Model. Journal of Thoracic Oncology. 2021;16(3 Supplement):S120.

33. Argillander TE, van der Zanden V, van der Zaag-Loonen HJ, Paarlberg KM, Meijer WJ, Kruse AJ, et al. Preoperative physical activity and frailty in older patients undergoing cancer surgery - PREsurgery study. Journal of Geriatric Oncology. 2022;13(3):384-7.

34. Buttery AK. Cardiac Rehabilitation for Frail Older People. Advances in Experimental Medicine and Biology. 2020;1216:131-47.

35. Czobor NR, Lehot J-J, Holndonner-Kirst E, Tully PJ, Gal J, Szekely A. Frailty In Patients Undergoing Vascular Surgery: A Narrative Review Of Current Evidence. Therapeutics & Clinical Risk Management. 2019;15:1217-32.

36. Daniel K. Wii-hab for pre-frail older adults. Rehabilitation nursing. 2012;37(4):195‐201.

37. Daniels S, Lee M, Moug S, Wilson T, Burton M, Brown S, et al. Semi-structured healthcare professional interviews to explore their preferences for the assessment and optimisation of older adults facing major gastrointestinal surgery. European Journal of Surgical Oncology. 2021;47(1):e25.

38. Ettema R, Schuurmans MJ, Schutijser B, van Baar M, Kamphof N, Kalkman CJ. Feasibility of a nursing intervention to prepare frail older patients for cardiac surgery: a mixed-methods study. European Journal of Cardiovascular Nursing. 2015;14(4):342-51.

39. Forcillo J, Perrault LP. If too frail, functional benefit following cardiac surgery may fail: A role for prehabilitation? The Journal of Thoracic and Cardiovascular Surgery. 2017;154(6):2000-1.

40. Gill TM, Baker DI, Gottschalk M, Peduzzi PN, Allore H, Van Ness PH. A prehabilitation program for the prevention of functional decline: effect on higher-level physical function. Archives of Physical Medicine and Rehabilitation. 2004;85(7):1043-9.

41. Goh SSN, Chia CL. Improving outcomes in geriatric surgery: Is there more to the equation? World Journal of Clinical Cases. 2022;10(13):4321-3.

42. Griebling TL. Re: Accumulated frailty characteristics predict postoperative discharge institutionalization in the geriatric patient. Journal of Urology. 2011;186(6):2304-5.

43. Lin FP, Bloomer PM, Grubbs R, Rockette-Wagner B, Tevar AD, Forman DE, et al. Low daily step count from a personal activity tracker is associated with a higher risk for hospital admission and death in community-dwelling patients with cirrhosis. Hepatology. 2021;74(SUPPL 1):1224A.

44. Pellathy TP, Rodriguez KL, Hruska KL, Kennedy KA, Hall DE. Shared Decision Making in High Risk Settings: Frail Patients' Perceptions of Preoperative Goal Clarification Consultation By Palliative Care Specialists. Journal of the American College of Surgeons. 2021;233(5 Supplement 1):S98-S9.

45. Puts MTE, Toubasi S, Andrew MK, Ashe MC, Ploeg J, Atkinson E, et al. Interventions to prevent or reduce the level of frailty in community-dwelling older adults: A scoping review of the literature and international policies. Age and Ageing. 2017;46(3):383-92.

46. Baimas-George M, Watson M, Thompson K, Shastry V, Iannitti D, Martinie JB, et al. Prehabilitation for Hepatopancreatobiliary Surgical Patients: Interim Analysis Demonstrates a Protective Effect From Neoadjuvant Chemotherapy and Improvement in the Frailty Phenotype. The American Surgeon. 2021;87(5):714-24.

47. Bojesen RD, Jørgensen LB, Grube C, Skou ST, Johansen C, Dalton SO, et al. Fit for Surgery-feasibility of short-course multimodal individualized prehabilitation in high-risk frail colon cancer patients prior to surgery. Pilot and Feasibility Studies. 2022;8(1):11.

48. Calabro M, Bonasso C, Pipitone Federico NS, Cuzzola B, Spidalieri L, Danna R, et al. A preoperative training program. Techniques in Coloproctology. 2020;24(4):352-3.

49. Carli F, Brown R, Kennepohl S. Prehabilitation to enhance postoperative recovery for an octogenarian following robotic-assisted hysterectomy with endometrial cancer. Canadian Journal of Anesthesia. 2012;59(8):779-84.

50. Carli F, Awasthi R, Gillis C, Kassouf W. Optimizing a frail elderly patient for radical cystectomy with a prehabilitation program. Canadian Urological Association Journal. 2014;8(11-12):E884-7.

51. Carli F, Bousquet-Dion G, Awasthi R, Elsherbini N, Liberman S, Boutros M, et al. Effect of multimodal prehabilitation versus postoperative rehabilitation on 30-day postoperative complications for frail patients undergoing resection of colorectal cancer: a randomized clinical trial [with consumer summary]. JAMA Surgery 2020 Mar;155(3):233-224. 2020.

52. Carli F, Bousquet-Dion G, Fiore JF. Prehabilitation vs Postoperative Rehabilitation for Frail Patients-Reply. JAMA Surgery. 2020;155(9):899-900.

53. Chia CLK, Mantoo SK, Tan KY. "Start to finish transinstitutional transdisciplinary care": A novel approach improves colorectal surgical outcomes in elderly patients. Annals of the Academy of Medicine Singapore. 2015;1):S126.

54. Cooper L, Frain L, Jaklitsch MT. Prehabilitation vs Postoperative Rehabilitation for Frail Patients. JAMA Surgery. 2020;155(9):898-9.

55. Derck J, Klemptner E, Mazurek A, Fakhoury J, Sonnenday C. Prehabilitation for patients with end-stage liver disease. American Journal of Transplantation. 2015;1):99.

56. Dholakia J, Cohn DE, Montemorano L, Straughn JM, Dilley SE. Prehabilitation is a cost-saving method with improved outcomes for medically frail patients undergoing surgery for epithelial ovarian cancer: A cost-effectiveness analysis. Gynecologic Oncology. 2020;159(Supplement 1):74.

57. Dworsky JQ, Castle SC, Lee CC, Singh SP, Russell MM. Gerofit Prehabilitation Pilot Program: Preparing Frail Older Veterans for Surgery. Journal for Healthcare Quality. 2019;41(2):91-8.

58. Ellenberger C, Schorer R, Bedat B, Hagerman A, Triponez F, Karenovics W, et al. How can we minimize the risks by optimizing patient's condition shortly before thoracic surgery? Saudi Journal of Anaesthesia. 2021;15(3):264-71.

59. Gillis C, Carli F. Patients with poor functional walking capacity experience significantly more medical complications post-colorectal surgery than those with higher functional walking capacity. European Journal of Surgical Oncology. 2021;47(5):1230-1.

60. Gillis C, Fenton TR, Gramlich L, Sajobi TT, Culos-Reed SN, Bousquet-Dion G, et al. Older frail prehabilitated patients who cannot attain a 400 m 6-min walking distance before colorectal surgery suffer more postoperative complications. European Journal of Surgical Oncology. 2021;47(4):874‐81.

61. Grimes L, Outtrim JG, Griffin SJ, Ercole A. Accelerometery as a measure of modifiable physical activity in high-risk elderly preoperative patients: a prospective observational pilot study. BMJ Open. 2019;9(11):e032346.

62. Gritsenko K, Helander E, Webb MPK, Okeagu CN, Hyatali F, Renschler JS, et al. Preoperative frailty assessment combined with prehabilitation and nutrition strategies: Emerging concepts and clinical outcomes. Best Practice and Research in Clinical Anaesthesiology. 2020;34(2):199-212.

63. Hanna K, Ditillo M, Joseph B. The role of frailty and prehabilitation in surgery. Current Opinion in Critical Care. 2019;25(6):717-22.

64. Ji YD, Dodson TB. Minimizing Frailty Preoperatively Through Prehabilitation: Improving Surgical Outcomes. Journal of Oral and Maxillofacial Surgery. 2022;80(1):6-7.

65. Kako J, Kajiwara K, Tatematsu N. Prehabilitation vs Postoperative Rehabilitation for Frail Patients. JAMA Surgery. 2020;155(9):897.

66. Kaplan JA, Brinson Z, Hofer R, O'Sullivan P, Chang A, Horvath H, et al. Early learners as health coaches for older adults preparing for surgery. Journal of Surgical Research. 2017;209:184-90.

67. Keller DS, Carter B, Moug SJ. Prehabilitation vs Postoperative Rehabilitation for Frail Patients. JAMA Surg. 2020;155(9):896.

68. Loh H, Foo F, Tan W, Sivarajah S, Chew M. Programme for enhanced elderly recovery at sengkang (PEERS). Diseases of the Colon and Rectum. 2020;63(6):e383.

69. Lorenz E, Hickson L, Weatherly R, Thompson K, Hogan M, Kennedy C. The impact of 4-weeks of supervised exercise on frailty and lower extremity (LE) function in patients with advanced chronic kidney disease (CKD). American Journal of Transplantation. 2021;21(SUPPL 4):318.

70. Magnano M, Andreis M, Nazionale G, Mola P, Machetta G. The role of prehabilitation in frail patient with a head and neck cancer. Oral Oncology. 2021;118(Supplement):3.

71. Milder DA, Pillinger NL, Kam PC. The role of prehabilitation in frail surgical patients: a systematic review. Acta Anaesthesiologica Scandinavica. 2018;62(10):1356-66.

72. Mrdutt MM, Papaconstantinou HT, Robinson BD, Bird ET, Isbell CL. Re: Preoperative frailty and surgical outcomes across diverse surgical subspecialties in a large health care system. Journal of Urology. 2019;202(4):637.

73. Ngo-Huang A, Fontillas RC, Gupta E, Sahai SK, Popovich S, Andrabi T, et al. Implementing prehabilitation as part of enhanced recovery after surgery (ERAS) efforts at a comprehensive cancer center: A team-based approach. Journal of Clinical Oncology Conference. 2018;36(30 Supplement 1).

74. Rafique H, Worley G, Anele C, Askari A, Faiz O. The use of prehabilitation in those undergoing colorectal surgery: A systematic review. Colorectal Disease. 2017;19(Supplement 2):68.

75. Ramírez-Martín R, Pérez-Rodríguez P, Menéndez-Colino R, Martín Maestre I, Gazo Martínez JA, Marijuán Martín JL, et al. Prehabilitation and perioperative geriatric care in patients aged over 80 years with colorectal cancer: Results of a cross-speciality geriatrics program. Journal of Geriatric Oncology. 2022.

76. Resnick MJ. Re: Effect of Multimodal Prehabilitation vs Postoperative Rehabilitation on 30-Day Postoperative Complications for Frail Patients Undergoing Resection of Colorectal Cancer: A Randomized Clinical Trial. J Urol. 2020;204(4):869-70.

77. Rostoft S. Improving the care for older patients with cancer. European Geriatric Medicine. 2015;1):S158-S9.

78. Salvi R, Meoli I, Cennamo A, Perrotta F, Saverio Cerqua F, Montesano R, et al. Preoperative high-intensity training in frail old patients undergoing pulmonary resection for NSCLC. Open Medicine (Poland). 2016;11(1):443-8.

79. Siu ATY, Poulton T, Ismail H, Riedel B, Dhesi J. Prehabilitation in the Older People: Current Developments. Current Anesthesiology Reports. 2021;11(4):373-80.

80. Söhle M, Coburn M. [Perioperative Medicine in Visceral Surgery in the Elderly Patient from an Anaesthesiological Perspective]. Zentralblatt für Chirurgie. 2021;146(3):296-305.

81. Van Der Hulst H, Bastiaannet E, van der Bol J, Dekker JW. Can prehabilitation protect frail colorectal cancer patients over 70 years old from complications after surgery? European Journal of Surgical Oncology. 2021;47(2):e22.

82. van der Hulst HC, Bastiaannet E, Portielje JEA, van der Bol JM, Dekker JWT. Can physical prehabilitation prevent complications after colorectal cancer surgery in frail older patients? European Journal of Surgical Oncology. 2021;47(11):2830-40.

83. van der Vlies E, Smits AB, Los M, van Hengel M, Bos WJW, Dijksman LM, et al. Implementation of a preoperative multidisciplinary team approach for frail colorectal cancer patients: Influence on patient selection, prehabilitation and outcome. Journal of Geriatric Oncology. 2020;11(8):1237-43.

84. Van Wijk L, Buis CI, Klaase JM. Feasibility of a prehabilitation clinic for patients undergoing oncologic abdominal surgery: the FRAIL study. European Journal of Surgical Oncology. 2020;46(2):e15.

85. Visina JM, Bloomer PM, Lin FP, Dunn MA, Josbeno DA, Tevar AD, et al. Prehabilitation adherence as predictor for improved physical function in cirrhosis. Hepatology. 2020;72(1 SUPPL):834A-5A.

86. Waite I, Deshpande R, Baghai M, Massey T, Wendler O, Greenwood S. Home-based preoperative rehabilitation (prehab) to improve physical function and reduce hospital length of stay for frail patients undergoing coronary artery bypass graft and valve surgery. Journal of Cardiothoracic Surgery. 2017;12(1):91.

87. Yau KW, Underwood MJ, Joynt GM, Lee A. Determinants of exercise intensity achieved during exercise prehabilitation program for patients awaiting cardiac surgery. Journal of the Hong Kong College of Cardiology. 2020;28(2):96.

88. Yau KWD, Underwood M, Joynt G, Lee A. Exercise prehabilitation (Prequel) for patients undergoing cardiac surgery: Preliminary results. Anesthesia and Analgesia. 2021;133(3 SUPPL 2):1485-6.

89. Cheng XS, Myers JN, Chertow GM, Rabkin R, Chan KN, Chen Y, et al. Prehabilitation for kidney transplant candidates: Is it time? Clinical Transplantation. 2017;31(8).

90. Lorenz EC, Hickson LJ, Weatherly RM, Thompson KL, Walker HA, Rasmussen JM, et al. Protocolized exercise improves frailty parameters and lower extremity impairment: A promising prehabilitation strategy for kidney transplant candidates. Clinical Transplantation. 2020;34(9):e14017.

91. Michalik C, Maciukiewicz P, Drewa T, Kenig J, Juszczak K. Frailty, geriatric assessment and prehabilitation in elderly patients undergoing urological surgery - is there a need for change of the daily clinical practice? Synthesis of the available literature. Central European Journal of Urology. 2020;73(2):220-5.

92. Oosting E, Hoogeboom T, Appelman S, Dronkers J, Van Meeteren N. Feasibility of an intensive therapeutic exercise program for frail elderly prior to total hip arthroplasty: Two randomized pilot studies. Physiotherapy (United Kingdom). 2015;1):eS1150-eS1.

93. Baimas-George M, Watson M, Elhage S, Parala-Metz A, Vrochides D, Davis BR. Prehabilitation in Frail Surgical Patients: A Systematic Review. World Journal of Surgery. 2020;44(11):3668-78.

94. McIsaac DI, Hladkowicz E, Bryson GL, Forster AJ, Gagne S, Huang A, et al. Home-based prehabilitation with exercise to improve postoperative recovery for older adults with frailty having cancer surgery: the PREHAB randomised clinical trial. British Journal of Anaesthesia. 2022.

95. van Munster B. PREHABILITATION IN PREOPERATIVE CARE OF PREHAB AS MEDICINE. Journal of Geriatric Oncology. 2019;10(6 Supplement 1):S4.

96. van Wijk L, van der Schnee L, Buis CI, Hentzen JEKR, Haveman ME, Klaase JM. Reengineering the Preoperative Care Path to Implement a Prehabilitation Outpatient Clinic for Patients Referred for Hepatobiliary and Pancreatic Surgery. HPB. 2021;23(Supplement 3):S1029.

97. Shovel L, Dunne J, Whibley J, Fernandes A, Kasivisvanathan R. A tertiary cancer centre experience of prehabilitation for surgical ovarian cancer patients receiving neoadjuvant chemotherapy: The royal Mile - Marsden integrated lifestyle and exercise programm. Clinical Nutrition ESPEN. 2019;31:138.

98. Actrn. A randomised clinical trial comparing preoperative exercise in the home, hospital, and community with standard care in adults awaiting for major abdominal surgery. https://trialsearchwhoint/Trial2aspx?TrialID=ACTRN12622000001796. 2022.

99. Janssen TL, Mosk CA, van Hoof-de Lepper C, Wielders D, Seerden TCJ, Steyerberg EW, et al. A multicomponent prehabilitation pathway to reduce the incidence of delirium in elderly patients in need of major abdominal surgery: study protocol for a before-and-after study. BMC Geriatrics. 2019;19(1):87.

100. Lindback Y, Tropp H, Enthoven P, Abbott A, Oberg B. PREPARE: pre-surgery physiotherapy for patients with degenerative lumbar spine disorder: a randomized controlled trial. The Spine Journal 2017 Aug;18(8):1347-1355. 2017.

101. Negm AM, Kennedy CC, Ioannidis G, Gajic-Veljanoski O, Lee J, Thabane L, et al. Getting fit for hip and knee replacement: a protocol for the Fit-Joints pilot randomized controlled trial of a multi-modal intervention in frail patients with osteoarthritis. Pilot and Feasibility Studies. 2018;4(1).

102. Ntr. Prehabilitation for bowel cancer patients undergoing surgery to improve fitness and reduce complications. https://trialsearchwhoint/Trial2aspx?TrialID=NTR5947. 2016.

103. Santa Mina D, Sellers D, Au D, Alibhai SMH, Clarke H, Cuthbertson BH, et al. A Pragmatic Non-Randomized Trial of Prehabilitation Prior to Cancer Surgery: Study Protocol and COVID-19-Related Adaptations. Frontiers in Oncology. 2021;11:629207.

104. Brown R, Carli F, Kennepohl S. Prehabilitation for a frail elderly patient with endometrial cancer: A case report. Canadian Journal of Anesthesia Conference. 2012;59(SUPPL. 1).

105. Carli F, Bousquet-Dion G, Awasthi R, Elsherbini N, Liberman S, Boutros M, et al. Effect of Multimodal Prehabilitation vs Postoperative Rehabilitation on 30-Day Postoperative Complications for Frail Patients Undergoing Resection of Colorectal Cancer: a Randomized Clinical Trial. JAMA surgery. 2020;155(3):233‐42.

106. Debes C, Aissou M, Beaussier M. [Prehabilitation. Preparing patients for surgery to improve functional recovery and reduce postoperative morbidity]. Annales Françaises d'Anesthésie et de Réanimation. 2014;33(1):33-40.

107. Liu C, Zhu M. Progress of researches on application of multimodal prehabilitation technique in geriatric surgery. [Chinese]. Chinese Journal of Clinical Nutrition. 2020;28(6):368-73.

108. Duerksen JR. Hip fractures: special considerations for the elderly patient. Orthopedic nursing / National Association of Orthopedic Nurses. 1982;1(1):11-9.

109. Eckmann L. Surgery in the old age (author's transl). Schweizerische Rundschau fur Medizin Praxis = Revue suisse de medecine Praxis. 1977;66(37):1183-7.

110. Rosenberg J. Plan ahead to improve surgical outcomes for the elderly. Medical Management Network. 1998;6(11):5-8.

111. Feng S, Dorrance K, Saunders C, Bryson G, Gagne S, Hladkowicz E, et al. Experience with exercise prehabilitation before cancer surgery for older people with frailty. Canadian Journal of Anesthesia. 2019;66(2):S309‐S10.

112. Bruns ERJ, Argillander TE, Schuijt HJ, van Duijvendijk P, van der Zaag ES, Wassenaar EB, et al. Fit4SurgeryTV At-home Prehabilitation for Frail Older Patients Planned for Colorectal Cancer Surgery: A Pilot Study. American Journal of Physical Medicine & Rehabilitation. 2019;98(5):399-406.

113. Singer JP, Soong A, Bruun A, Hays S, Kukreja J, Bracha A, et al. "pre-habilitation" of frail candidates for lung transplantation using a mobile health enabled home-based intervention is feasible and safe. American Journal of Respiratory and Critical Care Medicine Conference: American Thoracic Society International Conference, ATS. 2017;195(no pagination).

114. Singer JP, Soong A, Bruun A, Bracha A, Chin G, Hays SR, et al. A mobile health technology enabled home-based intervention to treat frailty in adult lung transplant candidates: A pilot study. Clinical Transplantation. 2018;32(6):e13274.

115. Balagué C, Arroyo A. Prehabilitation: Another step towards the optimization of surgical patients. Cirugía Española (English Edition). 2020;98(4):175-7.

116. Agasi-Idenburg C, Koning-van Zuilen M, Stuiver M, Punt C, Aaronson N, Westerman M. Preferences, barriers and facilitators for preoperative exercise participation for elderly treated for colorectal cancer and their social network. Supportive Care in Cancer. 2018;26(2 Supplement 1):S61.

117. Agasi-Idenburg CS, Zuilen MK, Westerman MJ, Punt CJA, Aaronson NK, Stuiver MM. "I am busy surviving" - Views about physical exercise in older adults scheduled for colorectal cancer surgery. Journal of Geriatric Oncology. 2020;11(3):444-50.

118. Johanning J, Hall D. Successful Prehabilitation: A Noble Goal. Journal of the American Geriatrics Society. 2018;66(10):1869.

119. Gill TM, Baker DI, Gottschalk M, Gahbauer EA, Charpentier PA, de Regt PT, et al. A prehabilitation program for physically frail community-living older persons. Archives of Physical Medicine and Rehabilitation. 2003;84(3):394-404.

120. McAdams-DeMarco MA, Ying H, Van Pilsum Rasmussen S, Schrack J, Haugen CE, Chu NM, et al. Prehabilitation prior to kidney transplantation: Results from a pilot study. Clinical Transplantation. 2019;33(1):e13450.

121. Boreskie KF, Hay JL, Kehler DS, Johnston NM, Rose AV, Oldfield CJ, et al. Prehabilitation: The Right Medicine for Older Frail Adults Anticipating Transcatheter Aortic Valve Replacement, Coronary Artery Bypass Graft, and Other Cardiovascular Care. Clinics in Geriatric Medicine. 2019;35(4):571-85.

122. Lin FP, Visina JM, Bloomer PM, Dunn MA, Josbeno DA, Zhang X, et al. Prehabilitation-Driven Changes in Frailty Metrics Predict Mortality in Patients With Advanced Liver Disease. American Journal of Gastroenterology. 2021;116(10):2105-17.

123. Gurlit S, Gogol M. Prehabilitation is better than cure. Current Opinion in Anesthesiology. 2019;32(1):108-15.

124. Perlmutter BC, Ali J, Cengiz TB, Said SA, Tang A, Augustin T, et al. Correlation between physical status measures and frailty score in patients undergoing pancreatic resection. Surgery. 2022;171(3):711-7.

125. Ng P, Lee JKD, Tan KY. Finding value with prehabilitation in older persons receiving surgery. Current Opinion in Supportive and Palliative Care. 2022;16(1):19-24.

126. Mohamed B, Ramachandran R, Rabai F, Price CC, Polifka A, Hoh D, et al. Frailty Assessment and Prehabilitation Before Complex Spine Surgery in Patients With Degenerative Spine Disease: A Narrative Review. Journal of Neurosurgical Anesthesiology. 2021.

127. Punt IM, van der Most R, Bongers BC, Didden A, Hulzebos EHJ, Dronkers JJ, et al. [Improving pre- and perioperative hospital care : Major elective surgery]. Bundesgesundheitsblatt Gesundheitsforschung Gesundheitsschutz. 2017;60(4):410-8.

128. Wang T, Stanforth PR, Fleming RYD, Wolf JS, Jr., Stanforth D, Tanaka H. A Mobile App With Multimodality Prehabilitation Programs for Patients Awaiting Elective Surgery: Development and Usability Study. JMIR Perioperative Medicine. 2021;4(2):e32575.

129. Arora RC, Brown CH, Sanjanwala RM, McKelvie R. "NEW" Prehabilitation: A 3-Way Approach to Improve Postoperative Survival and Health-Related Quality of Life in Cardiac Surgery Patients. Canadian Journal of Cardiology. 2018;34(7):839-49.

130. Jensen BT, Lauridsen SV, Scheede-Bergdahl C. The Potential of Prehabilitation in Radical Cystectomy Pathways: Where Are We Now? Seminars in Oncology Nursing. 2021;37(1):151107.

131. Shovel L, Morkane C. Prehabilitation for Vascular Surgery Patients: Challenges and Opportunities. Canadian Journal of Cardiology. 2022;38(5):645-53.

132. Rumer KK, Saraswathula A, Melcher ML. Prehabilitation in our most frail surgical patients: are wearable fitness devices the next frontier? Current Opinion in Organ Transplantation. 2016;21(2):188-93.

133. Bongers BC, Klaase JM, van Meeteren NLU. Prehabilitation vs Postoperative Rehabilitation for Frail Patients. JAMA Surgery. 2020;155(9):896-7.

134. Waterland JL, Ismail H, Riedel B. Prehabilitation vs Postoperative Rehabilitation for Frail Patients. JAMA Surgery. 2020;155(9):897-8.

135. Carli F, Bessissow A, Awasthi R, Liberman S. Prehabilitation: finally utilizing frailty screening data. European Journal of Surgical Oncology. 2020;46(3):321-5.

136. Oosting E, Jans MP, Dronkers JJ, Naber RH, Dronkers-Landman CM, Appelman-de Vries SM, et al. Preoperative home-based physical therapy versus usual care to improve functional health of frail older adults scheduled for elective total hip arthroplasty: a pilot randomized controlled trial. Archives of Physical Medicine and Rehabilitation. 2012;93(4):610-6.

137. Hoogeboom TJ, Dronkers JJ, van den Ende CH, Oosting E, van Meeteren NL. Preoperative therapeutic exercise in frail elderly scheduled for total hip replacement: a randomized pilot trial. Clinical Rehabilitation. 2010;24(10):901-10.

138. Williams FR, Berzigotti A, Lord JM, Lai JC, Armstrong MJ. Review article: impact of exercise on physical frailty in patients with chronic liver disease. Alimentary Pharmacology and Therapeutics. 2019;50(9):988-1000.

139. Yau DKW, Underwood MJ, Joynt GM, Lee A. What demographic factors influence participation in a randomised controlled trial on prehabilitation for cardiac surgery? Journal of the Hong Kong College of Cardiology. 2020;28(1):19.

140. Durand MJ, Beckert AK, Peterson CY, Ludwig KA, Ridolfi TJ, Lauer KK, et al. You Are Only as Frail as Your Arteries: Prehabilitation of Elderly Surgical Patients. Current Anesthesiology Reports. 2019;9(4):380-6.

141. Furyk C, Senthuran S, Nye D, Ho YH, Leicht AS. Prehabilitation for Frail Patients Undergoing Colorectal Surgery: Lessons Learnt From a Randomised Feasibility Study. Frontiers in Rehabilitation Sciences. 2021;2:650835.

142. Afilalo J. Evaluating and treating frailty in cardiac rehabilitation. Clinics in Geriatric Medicine. 2019;35(4):445-57.

143. Heil TC, Driessen EJ, Argillander TE, Melis RJ, Maas HA, Olde Rikkert MG, et al. Implementation of prehabilitation in colorectal cancer surgery: qualitative research on how to strengthen facilitators and overcome barriers. Supportive Care in Cancer. 2022:1-14.

144. Yau KW, Tsang WWN. Enhancing Postoperative Recovery – Prehabilitation for Frail Patients Undergoing Cardiac Surgery [Ph.D.]. Ann Arbor: The Chinese University of Hong Kong (Hong Kong); 2021.

## Appendix H: Information on funding and conflict of interest

| Document | Year | Funding | Conflict of interest |
| --- | --- | --- | --- |
| Feng et al. (111) | 2019 | Not reported | Not reported |
| Bruns et al. (112) | 2019 | None | None |
| Singer et al. (113) | 2017 | Nina Ireland Program in Lung Health Award | Not reported |
| Singer et al. (114) | 2018 | National Heart, Lung, and Blood Institute; Nina Ireland Program in Lung Health Award | one of the authors is CEO of the company Aidcube, which offers pulmonary rehabilitation services for COPD patients and is used in the study |
| Balagué & Arroyo (115) | 2020 | Not reported | Not reported |
| Agasi-Idenburg et al. (116) | 2018 | Not reported | Not reported |
| Agasi-Idenburg et al. (117) | 2020 | grant by the Netherlands Organization for Scientific Research | None |
| Johanning & Hall (118) | 2018 | Not reported | "Dr. Johanning reports owning intellectual property through FUTUREASSURE LLC. Dr. Hall reports a consulting position with University of Pittsburgh Medical Centers" |
| Gill et al. (119) | 2003 | grant from the Claude D. Pepper Older Americans Independence Center from the National Institute on Aging and the Gaylord Rehabilitation Research Institute and by the National Institute on Aging | “No commercial party having a direct financial interest in the results of the research supporting this article has or will confer a benefit upon the author(s) or upon any organization with which the author(s) is/are associated.“ |
| McAdams-DeMarco et al. (120) | 2019 | support from the Mendez National Institute for Transplantation Foundation, Johns Hopkins University Claude D. Pepper Older Americans Independence Center | “The authors of this manuscript declare no conflict of interest.“ |
| Boreskie et al. (121) | 2019 | Not reported | “R.C. Arora has received honoraria from Mallinckrodt Pharmaceuticals and Abbott Nutrition and an unrestricted educational grant from Pfizer Canada unrelated to this article. The other authors have nothing to disclose.” |
| Lin et al. (122) | 2021 | one author was supported by a scholarship grant for study extension abroad, sponsored by the Spanish Association for the Study of the Liver (AEEH) | None |
| Gurlit & Gogol (123) | 2019 | None | None |
| Perlmutter et al. (124) | 2022 | None | None |
| Ng et al. (125) | 2022 | None | None |
| Mohamed et al. (126) | 2021 | Not reported | None |
| Punt et al. (127) | 2017 | Not reported | None |
| Wang et al. (128) | 2021 | Not reported | None |
| Arora et al. (129) | 2018 | Not reported | all but one of the authors have received financial contributions from medical firms unrelated to this research as well as research grants from national institutes |
| Jensen et al. (130) | 2021 | None | Not reported |
| Shovel & Morkane (131) | 2022 | None | None |
| Rumer et al. (132) | 2016 | all authors received financial support by the Stanford Society of Physician Scholars | None |
| Bongers et al. (133) | 2020 | Not reported | None |
| Waterland (134) | 2020 | Not reported | None |
| Carli et al. (135) | 2020 | Not reported | Not reported |
| Oosting et al. (136) | 2012 | supported by the Scientific College Physical Therapy of the Royal Dutch Society for Physical Therapy | Not reported |
| Hoogeboom et al. (137) | 2010 | Hospital Gelderse Vallei, Ede, The Netherlands | None |
| Williams et al. (138) | 2019 | None | None |
| Yau et al. (139) | 2020 | Not reported | Not reported |
| Durand et al. (140) | 2019 | Not reported | None |
| Furyk et al. (141) | 2021 | Townsville Hospital and Health Service Research Trust Fund, Australian and New Zealand College of Anaesthetists (ANZCA) Novice investigator grant | “The authors declare that the research was conducted in the absence of any commercial or financial relationships that could be construed as a potential conflict of interest.“ |
| Afilalo (142) | 2019 | supported by grants from the Canadian Institutes of Health Research, the Heart and Stroke Foundation of Canada, and the Fonds de recherche du Québec en Santé. | Not reported |
| Heil et al. (143) | 2022 | Zorgevaluatie Leading the Change grant | “The authors declare no competing interests.“ |
| Yau (144) | 2021 | Not reported | Not reported |

## Appendix I: Relevance and Rigor

| **References** | **Study Design** | **Rigor of study design** | **Real-life environment (relevance)** |
| --- | --- | --- | --- |
| Afilalo (142) | Narrative review/ perspective article | - | Generalized, not context-specific due to review format |
| Agasi-Idenburg et al. (116) | Qualitative, exploratory study | + | Real-life experiences by patients, informal caregivers, and healthcare professionals |
| Agasi-Idenburg et al. (117) |  |  |  |
| Arora et al. (129) | Narrative review | o | Generalized, not context-specific due to review format |
| Balagué &  Arroyo (115) | Editorial | - | Generalized, not context-specific, based on authors’ experience and knowledge |
| Bongers et al. (133) | Letter to the editor | - | Generalized, not context-specific, based on authors’ experience and knowledge |
| Boreskie et al. (121) | Review | o | Generalized, not context-specific due to review format |
| Bruns et al. (112) | Non-randomized pilot observational study | + | Experimental but non-controlled setting |
| Carli et al. (135) | Narrative review/ perspective article | o | Generalized, not context-specific due to review format |
| Durand et al. (140) | Review | o | Generalized, not context-specific due to review format |
| Feng et al. (111) | Nested qualitative study within an RCT | + | Insights from a controlled study setting |
| Furyk et al. (141) | Randomized, controlled feasibility study | o | Insights from a controlled study setting |
| Gill et al. (119) | Demonstration study | + | Part of study, however setting in general community |
| Gurlit & Gogol (123) | Review | o | Generalized, not context-specific due to review format |
| Heil et al. (143) | Qualitative study | + | Real-life experiences by different healthcare professionals |
| Hoogeboom et al. (137) | Single-blind, randomized clinical pilot trial | o | Insights from a controlled study setting |
| Jensen et al. (130) | Narrative review | o | Generalized, not context-specific due to review format |
| Johanning & Hall (118) | Editorial | - | Generalized, not context-specific, based on authors’ experience and knowledge |
| Lin et al. (122) | Cohort study | + | Insights from a controlled study setting |
| McAdams- DeMarco et al. (120) | Single-arm intervention pilot study | o | Insights from a controlled study setting |
| Mohamed et al. (126) | Review | o | Generalized, not context-specific due to review format |
| Ng et al. (125) | Review | o | Generalized, not context-specific due to review format |
| Oosting et al. (136) | Single-blind pilot randomized controlled trial | o | Insights from a controlled study setting |
| Perlmutter et al. (124) | Observational study | + | Insights from a controlled study setting |
| Punt et al. (127) | Narrative review/perspective article | o | Generalized, not context-specific due to review format |
| Rumer et al. (132) | Narrative review | o | Generalized, not context-specific due to review format |
| Shovel & Morkane (131) | Narrative review | o | Generalized, not context-specific due to review format |
| Singer et al. (113) | Non-randomized, observational pilot study | + | Insights from a controlled study setting |
| Singer et al. (114) | Non-randomized, observational pilot study |  |  |
| Wang et al. (128) | pilot observational study | + | Insights from a controlled study setting |
| Waterland et al. (134) | Letter to the editor | - | Generalized, not context-specific, based on authors’ experience and knowledge |
| Williams et al. (138) | Review | o | Generalized, not context-specific due to review format |
| Yau (144) | RCT and systematic review | o | Insights from a controlled study setting |
| Yau et al. (139) | stratified randomized controlled trial | o |  |

+ = positive, e.g. observational studies in a real health system setting as well as qualitative interviews that provide first-hand information what affects the implementation process that are usually context-specific

- = negative, e.g. information from editorials or opinion pieces, in which the quality of the information can vary greatly with the expertise of the author

o = neutral, e.g. systematic reviews that provide more generalized information
